# Supplementary material for: A scoping review of recommendations in the English language on conducting research with trauma-exposed populations since publication of the Belmont report; thematic review of existing recommendations on research with trauma-exposed populations
Source: PLoS One. 2021 Jul 29;16(7):e0254003. doi: 10.1371/journal.pone.0254003 (PMC8321367; doi:10.1371/journal.pone.0254003)
Supplement: S1 File — (DOCX) [file pone.0254003.s001.docx]

Search String Used:

(Trauma OR Trauma-informed OR Trauma-focused OR “Survivor of trauma” OR “Survivors of trauma” OR “Trauma survivor” OR Survivor OR “Special population” OR “Sensitive subject” OR “Sensitive topic” OR “Vulnerable population” OR Vulnerable OR “Vulnerable group” OR Retraumatization) AND (Guidelines OR Principles OR Framework OR Protocol) AND (Ethics OR Ethical OR “Clinical practice guidelines” OR “Universal trauma screening” OR Retraumatization OR “Trauma research design” OR “Trauma informed interviewing” OR “Trauma informed focus groups” OR “Trauma informed survey design” OR  “Safety protocols” OR “Distress protocols” OR “Participant reactions”) AND NOT (“brain trauma” OR “neurotrauma” OR “fracture” OR “traumatic brain injury”)

**Selected Recommendations Pertaining to Community Benefit by Article**

| **Article** | **Publication Type** | **Study Type** | **Population^[[1]](#endnote-1)^** | **When Trauma Occurred** | **Trauma Type** | **Country** | **Recommendations** |
| --- | --- | --- | --- | --- | --- | --- | --- |
| Ahrens, Isas, & Viveros (2011)[1] | Peer reviewed article | Qualitative | Latina women | Adulthood | Sexual | United States | Have your research team mirror the community. Train researchers on what is culturally appropriate and what is not. Spend time in the community before recruitment and use community-grounded recruitment methods. Use participatory research approaches and consider the benefits of qualitative methods for disclosure. |
| Allodi (1991) [2] | Peer reviewed article | Review | Not applicable | Childhood and adulthood | Torture | Multiple | Incorporate complexity in instruments to study torture survivors. If using short screening tools (e.g., in a larger population-based survey), have a subset take a longer assessment to validate the tool. Use standardized measures to facilitate cross-study comparisons. Continue research on torture survivors. |
| Alvarez (2020) [3] | Peer reviewed article | Review | Students | Childhood | Not specified | United States | Incorporate analysis of racism into your research. |
| Anderson, Craig, & Ziedonis (2017) [4] | Peer reviewed article | Qualitative | Deaf individuals with trauma exposure | Childhood and adulthood | Developmental/adverse childhood experiences (ACEs), emotional, natural disaster, physical, sexual, terrorism/war | United States | Use community based participatory research (CBPR)methods [5] ^[[2]](#endnote-2)^ |
| Andrews, Pepler, & Motz (2019) [6] | Peer reviewed article | Not research | Women engaging with community-based organizations | Not specified | Intimate partner violence | Canada | Do relational and trauma informed research. Trauma informed research engages with community partners and is safety-focused. Relational research supports relationships within and between researcher and community partner groups, and across sectors. The study protocol should address the following elements: “aligning values, valuing community perspectives, fostering trust and openness, addressing issues of power inequity, ensuring research components are useful to communities, addressing issues of confidentiality and information use.” (p.550) The following strategies can be used: “developing awareness, considering project readiness, engaging and building relationships, supporting the training of facilitators in research and evaluation, embedding relational and trauma-informed approaches in research, providing ongoing mentoring for sustainability, ensuring project capacity for trauma-informed work.”(p.551) |
| Avey et al. (2018) [7] | Peer reviewed article | Qualitative | American Indians and Alaska Natives | Not specified | Not specified | United States | Disseminate results widely, focusing dissemination to community members. Give a summary update for each research phase. Apply results to improve trauma care. |

**Selected Recommendations Pertaining to Community Benefit by Article**

| **Article** | **Publication Type** | **Study Type** | **Population^i^** | **When Trauma Occurred** | **Trauma Type** | **Country** | **Recommendations** |
| --- | --- | --- | --- | --- | --- | --- | --- |
| Barrios et al. (2020) [8] | Peer reviewed article | Qualitative | Mothers who had left an abusive partner | Adulthood | Intimate partner violence | United States | Ask questions that examine stress and discrimination with an intersectional lens (Crenshaw, 1989) [9]. Explore how expectations and norms about gender, family, and intimate partner violence affect participants’ views on leaving an abusive relationship. Engage participants on how they feel expectations and norms about gender and relationships may differ if the participant belonged to different demographic groups, how they identify, how they view intimate partner violence and how their view relates to others’ views, how they feel authorities treat survivors, what resources are available to them, how the providers of resources treat them based on social status and facilitators and barriers to accessing resources |
| Beharry et al. (2018) [10] | Organizational report, position paper | Not research | Homeless and migrant youth | Childhood | Emotional, physical, sexual, terrorism/war | Not applicable | Address stigma. Consider ways to foster participant involvement throughout research. Conduct research with policy and intervention design in mind. Encourage cross-sector collaboration to address needs. |
| Benevides et al. (2020) [11] | Peer reviewed article | Mixed methods | Autistic adults | Not specified | Not specified | United States, Australia, Russia | Researchers should examine (list from a group of autistic adults): “1. What is the impact of trauma on mental health outcomes in autistic individuals, and what approaches can be used to effectively address trauma among autistic adults (e.g. trauma-informed care)? What are the best indicators or measures of PTSD^[[3]](#endnote-3)^, trauma, and adverse childhood experiences in autistic individuals? 2. What is the impact of social isolation, stigma, discrimination and other forms of marginalization on mental health and well-being in autistic individuals? Conversely, what is the impact of radical inclusion, such as being part of a social movement, on mental health and well-being? 3. When, for who, and under what conditions do self-managed interventions and preferred activities used to address well-being and mental health result in improved quality of life and reduced mental health symptoms? What is the effect of employing community available approaches and techniques such as exercise/physical activity, yoga, mindfulness and meditation, tai-chi, animal-assisted therapy, art and music-based approaches to well-being? 4. What are the potential long- and short-term negative side effects or adverse outcomes of currently recommended therapies and interventions (including behavioral and pharmacological), as measured in autistic individuals across the life span? 5. How can we develop better measurement tools for autistic quality of life, depression, anxiety, social well-being, and sleep as experienced by autistic adults?” (p.828) |

**Selected Recommendations Pertaining to Community Benefit by Article**

| **Article** | **Publication Type** | **Study Type** | **Population^i^** | **When Trauma Occurred** | **Trauma Type** | **Country** | **Recommendations** |
| --- | --- | --- | --- | --- | --- | --- | --- |
| Caldwell et al. (2005) [12] | Organizational report | Not research | American Indians and Alaska Natives | Childhood and adulthood | Not specified | United States | Use CBPR [5]^ii^ methods. Focus on specific communities rather than entire cultural groups. Take time to learn about the historical and current factors that affect the community you are working in and seek to have cultural humility.[13] Seek interventions that are culturally derived and honor re-traditionalization.[14] Include community members in all aspects of the research process though maintain awareness of the risks to confidentiality. Give communities ownership over results and data early in the research process. |
| Chamberlain et al. (2019) [15] | Peer reviewed article | Mixed methods, action research and review | Aboriginal parents with complex trauma, Aboriginal communities, and elders | Not defined | Historical/systemic trauma, individual-level trauma not specified | Australia | Intervention mapping and action research or community based participatory research are a strong combination to address trauma among Aboriginal populations. |
| Downes, Kelly, & Westmarland (2014) [16] | Guidelines, Editorial | Not research | Not applicable | Childhood and adulthood | Not specified | United Kingdom | Respect core values for trauma research: seeing people who have survived trauma (and perpetrators) as having agency, supporting participants (those who have experienced abuse and abusers) to make positive changes, and creating chances to increase benefits of participation and research influence. Develop research procedures with community partners to enhance buy-in but make sure that you also inform participants of what independence you have from community partners. Let participants know where the funding for the study is coming from. Consider impact of both conducting and choosing not to conduct a study on potential participants and communities. |
| Ghabarpour et al. (2018) [17] | Peer reviewed article | Qualitative | Intimate partner violence service providers | Not specified | Not specified | United States | Focus on the most affected. Support non-English speakers by ensuring access to resources, services, and research in multiple languages. Be trauma-aware during research conduct. Address history between the community and researchers. Create agreements and values with community. Empower community researchers. Focus on building upon community assets. Respect and recognize community sources of knowledge and values. |
| Hamby, Elm, & Schultz (2020) [18] | Peer reviewed article | Review | American Indian and Alaska Native elders | Childhood and adulthood | Historical/systemic | United States | Incorporate inquiry on historical trauma and how it interacts with other types of trauma. Consider creating measures of historical trauma using already existing data. Work in partnership with communities and recognize resilience. |

**Selected Recommendations Pertaining to Community Benefit by Article**

| **Article** | **Publication Type** | **Study Type** | **Population^i^** | **When Trauma Occurred** | **Trauma Type** | **Country** | **Recommendations** |
| --- | --- | --- | --- | --- | --- | --- | --- |
| Hebenstreit & DePrince (2012)(19) | Peer reviewed article | Experimental | Women reporting intimate partner abuse by male partners to police in a large United States city | Adulthood | Intimate partner violence | United States | Don’t exclude participants with trauma from participating in research. |
| Holmes et al. (2020) [20] | Peer- reviewed article | Not research | Global population affected by coronavirus | Childhood and adulthood | Pandemic | Focused on United Kingdom but applies global | Do interdisciplinary work on coronavirus. Share data and protocols, via existing research infrastructure when possible, to reduce needless duplication. Utilize CBPR [5]^ii^ methods. |
| Javakhishvili et al. (2020) [21] | Position paper | Not research | European population affected by coronavirus | Childhood and adulthood | Pandemic | European countries | Research how coronavirus affects medical provider mental health, family members of those lost to coronavirus, refugees and trafficked individuals, and how trauma-informed organizations may prevent occupational related burn-out. |
| Leung & Flanagan (2019) [22] | Peer- reviewed article | Not research | LGBTQ+ youth^[[4]](#endnote-4)^ | Childhood | Not specified | Not specified | Use photovoice and mobile interviewing as empowering research methodologies. |
| Leung et al. (2019) [23] | Peer reviewed article | Not research | Not specified | Childhood and adulthood | Not specified | Multiple countries | Focus on collaboration, building capacity, and working with existing movements (be collaborative and participatory). Seek to change harmful norms and hierarchies (be transformative). Be intersectional [9]. Engage in ongoing examination and modification of the research process as needed, seek to reduce risk and be accountable to community (be accountable). Share results freely and in accessible forums (be accessible and open). Use CBPR. [5]^ii^. Get training on vicarious trauma and power dynamics, including how to collect data from perpetrators in a way that facilitates sharing but does not condone the participant’s actions. Co-present findings with local researchers. Researchers from higher-income countries should seek and support collaborative learning environments with researchers from lower-income countries. |
| McCauley et al. (2019) [24] | Peer reviewed article | Not research | Not specified | Childhood and adulthood | Sexual | Not specified | Use transformative justice as research methodology when working with survivors and research the workings of transformative justice. Use post-traumatic growth as a minimum measure of success. Apply tenants of CBPR [5]^ii^. |

**Selected Recommendations Pertaining to Community Benefit by Article**

| **Article** | **Publication Type** | **Study Type** | **Population^i^** | **When Trauma Occurred** | **Trauma Type** | **Country** | **Recommendations** |
| --- | --- | --- | --- | --- | --- | --- | --- |
| Nnawulezi et al. (2019) [25] | Peer reviewed article | Mixed methods | Staff and clients at an organization serving survivors of torture, sexual assault, sex trafficking, and domestic violence | Not specified | Domestic violence, sexual, torture | United States | Attend to values of power sharing, relationships, recognizing expertise of community partners, and valuing personal and organizational growth. Attending to power sharing means that before a project commences research design, data ownership, and expectations regarding participation are agreed. Attending to relationships means learning as much as you can about context or potential partners through background material and events, having introductions and interaction, and honoring potential partners’ time and interest. Recognizing the expertise of community partners means co-designing research to the extant it is feasible, joint interpretation of data, and member checking. Valuing personal and organizational growth means using research to support improvement and empowerment. Specifically: “ensure that the community agency embodies organizational values of shared participation and power,” (p. 4832) that “participatory facilitation skills will strengthen relationships and result in shared knowledge,” “prioritize community partners’ needs over research needs,” (p. 4832) and “build participatory processes that rely on experiences rather than roles.” (p.4833) |
| Quina et al. (1999) [26] | Peer reviewed article | Mixed methods (survey and focus group) | Adults pursuing continuing education; New England university female students, convenience sample | Childhood and adulthood | Emotional, physical, sexual, | United States | Ground your research in ethical goals and a commitment to community. Seek input on your instruments from community members. |

**Selected Recommendations Pertaining to Community Benefit by Article**

| **Article** | **Publication Type** | **Study Type** | **Population^i^** | **When Trauma Occurred** | **Trauma Type** | **Country** | **Recommendations** |
| --- | --- | --- | --- | --- | --- | --- | --- |
| Roche et al. (2020) [27] | Peer reviewed article | Qualitative | People with lived experience of a mental health condition and First Nations, Metis, and immigrant people | Not specified | Not specified | Canada | Use a trauma-informed framework to engage community members in research. Components of this framework include trust, self-awareness, understanding and acceptance, relationship-building, and knowledge sharing, education, and communication. Foster trust by using a strengths-based perspective, openness and follow-up, and recognizing tenants of ownership, access, control, and possession. Use trauma-informed principles, be willing to challenge your biases and invest time in communities and have your own support systems in place to practice self-awareness, or reflexivity. Use storytelling and listening, cognitive behavioral therapy, and a strengths-based empowerment perspective to support understanding and acceptance. Relationship-building requires time, contact, and willingness to share. Foster knowledge sharing, education, and communication by transparency, early engagement with community, and member-checking. |
| Thomas, Weber, & Bradbury-Jones (2020) [28] | Peer reviewed article | Review | Populations in the Global South | Childhood and adulthood | Gender-based violence | Not indicated | Optimize the safety of participants and researchers, address power inequities within research, engage local systems within research ethics structures, and respect cultural context by, for instance, seeking community consent to conduct research. |
| Turpel-Lafond & Chondoma (2019) [29] | Organizational report | Qualitative | Indigenous and non-indigenous people working on information or research on Indigenous issues | Childhood and adulthood | Historical/systemic | Canada | Support Indigenous research done ‘inside-out,’ be Indigenous-centered, and be responsive to needs defined by Indigenous people and Nations. Build genuine relationships, focus on improving the lives of community members and yourself, and address one’s own (self or institutional) role in colonialism/racism. |

**Selected Recommendations Pertaining to Community Benefit by Article**

| **Article** | **Publication Type** | **Study Type** | **Population^i^** | **When Trauma Occurred** | **Trauma Type** | **Country** | **Recommendations** |
| --- | --- | --- | --- | --- | --- | --- | --- |
| Twis & Preble (2020) [30] | Peer reviewed article | Not research | Adults who were sex trafficked and now work as antitrafficking advocates | Not specified | Sexual | United States | Use intersectional standpoint methodology, which merges the strengths of intersectionality (Crenshaw, 1989) [9] and standpoint theory to do impactful research. Intersectionality (Crenshaw, 1989) [9] is a powerful framework for understanding the nuances of participants’ lives, if researchers can think intersectionally and practice reflexivity, but it does not offer a specific methodology. Standpoint theory is reductionist but proposes a methodology of working with participants, key informant interviews, and application of findings to social change. Consider the many social identities of participants and their lived experiences, the political-structural obstacles they face, and a target system for research to change. Use CBPR [5].^ii^ |
| Wright, Olomi, & DePrince (2020) [31] | Peer reviewed article | Mixed methods | Crime survivors | Not specified | Not specified | United States | Use community-engaged research for trauma research because it is political, values all voices, creates better quality data, undertakes research translation as an inherent part of the research process, and is an avenue for change. |

**Selected Recommendations Pertaining to Participant Benefit by Article**

| **Article** | **Publication Type** | **Study Type** | **Population^i^** | **When Trauma Occurred** | **Trauma Type** | **Country** | **Recommendations** |
| --- | --- | --- | --- | --- | --- | --- | --- |
| Burgess-Proctor (2015) [32] | Non-peer reviewed article | Not research | Women who experienced violent victimization | Childhood and adulthood | Emotional, physical, sexual | United States | Let participants select pseudonyms. End interviews with mention of participant strength and insight and offer to share results with participants. |
| Campbell, Goodman-Williams & Javorka (2019) [33] | Peer reviewed article | Not research | Participants in sexual violence research | Childhood and adulthood | Sexual | Not specified | Anticipate hearing about diverse trauma experiences, coping responses, and impacts n order to recognize the impact of trauma on participants. To help participants recover, offer referrals to local and culturally-competent resources. To support agency, give participants choice as to how their data will be presented. Also use CBPR [5]^ii^ methods. |
| Gekoski, Gray, & Adler (2012) [34] | Peer reviewed article | Qualitative | Women who have lost someone to homicide | Not specified | Vicarious/ secondary | United Kingdom | To increase the potential positive outcomes from research participation, don’t rush participants or break their flow of thought, co-guide interviews with participants, invest in rapport, and listen empathetically. Use research to affect change. Share results with participants. |
| Logan et al. (2008) [35] | Peer reviewed article | Qualitative | Rural and urban women who had experienced partner violence | Adulthood | Physical | United States | Ensure participants understand the study objectives and process. Focus on participant comfort; this may include being flexible with interviewing and willing to visit participants at home. |
| Paton, Horsfall, & Carrington (2018) [36] | Peer reviewed article | Qualitative | People connected to the Partners in Recovery (PIR) initiative in Western New South Wales | Not applicable | Not specified | Australia | Foster the recovery principles of connection, agency, exploration, belief that things can be better, empowerment, and de-stigmatization throughout the research process. Maximize supportive interactions. Consider participants as experts in their own lives and give them power and autonomy. Give participants information in multiple formats in each interaction and ask for their consent to continue involvement in the study each time. Create opportunities for participants to share their experiences in empowering and socially supported ways. |
| Pk (2018) [37] | Peer reviewed article | Not research | Homeless or unstably housed youth of color | Childhood | Developmental/ adverse childhood experiences (ACEs), historical/ systemic | United States | Research participation can be a positive experience for participants where they feel heard and recognized for their knowledge. Ensure that you use a site that is safe and accessible to participants. Consider allowing children or support animals. Learn about how participants cope and re-evaluate beliefs about activities or experiences that you may have stigmatized or not understood. Consider innovative ways to share results with participants who may experience much instability in their lives and may not have consistent contact information. |
| Raghavan & Sandanapitchai (2020) [38] | Peer reviewed article | Review | Individuals who have experienced a Criterion A^[[5]](#endnote-5)^ trauma(39) | Childhood and adulthood | Criterion A trauma(39) | Not specified | Culture can promote resilience through coping or meaning-making and should be inquired about in trauma research. |

**Selected Recommendations Pertaining to Participant Benefit by Article**

| **Article** | **Publication Type** | **Study Type** | **Population^i^** | **When Trauma Occurred** | **Trauma Type** | **Country** | **Recommendations** |
| --- | --- | --- | --- | --- | --- | --- | --- |
| Wager (2012) [40] | Peer reviewed article | Mixed methods | Adults and young adults (16+) | Childhood and adulthood | Sexual | United Kingdom | Participants may benefit from trauma research by being able to tell their story, feeling that they are contributing to change and helping others, having opportunities for reflection, de-stigmatization, and empowerment. Provide resources for participants to learn more about the topic and understand how study results will be used. In your debriefing, provide a summary of the literature on the topic in an accessible manner, including the prevalence of the trauma, and how results will be used. Include information on how people respond to trauma so that participants can better understand themselves and that their reactions to their experiences are appropriate. This will help destigmatize the trauma and participant reactions. |

**Selected Recommendations Pertaining to Safety by Article**

| **Article** | **Publication Type** | **Study Type** | **Population^i^** | **When Trauma Occurred** | **Trauma Type** | **Country** | **Recommendations** |
| --- | --- | --- | --- | --- | --- | --- | --- |
| Ahlin (2019) [41] | Peer reviewed article | Review | Incarcerated youth | Childhood | Developmental/ adverse childhood experiences (ACEs), sexual | United States | When seeking research assent from incarcerated youth be cautious that they may feel obligated to participate due to their restrictive and controlled environment, and they may also feel that participation could benefit them or their case. |
| Allard et al. (2019) [42] | Peer reviewed article | Qualitative | Japanese undergrad students | Childhood and adulthood | Developmental/ adverse childhood experiences (ACEs), natural disaster, physical, sexual | Japan | Anticipate that it can be culturally acceptable to ask about trauma in research conducted in collectivist-oriented cultures. |
| Allden et al. (2009) [43] | Peer reviewed article | Not research | Complex emergency settings | Childhood and adulthood | Not specified | Multiple | Have support available for participants that is culturally and politically applicable. Avoid stigmatization. Consider whether gathering for the research may make participants a target. Change research protocols, including inform consent, to adapt to changing circumstances. Get consent if participants may be identifiable in results (e.g., photos). Be aware of power dynamics that may affect participant consent. Be aware that participants may have expectations about research outcomes that are not realistic. Discuss research objectives and possible benefits multiple times so that participants can have realistic expectations. Consider the positive and negative impacts that results dissemination may have for participants. Check in with participants on findings before sharing. |
| Black et al. (2006) [44] | Peer reviewed article | Cross-sectional | Adults in the United States | Not specified | Intimate partner violence, physical, sexual | United States | Anticipate potential adverse reactions and try to minimize them. Ask participants if it is safe to ask them questions about violence. Have protocols in place to respond to participant distress. Provide participants with referrals for handling trauma. |
| Boscarino et al. (2004) [45] | Peer reviewed article | Cross-sectional | New York City residents | Adulthood | Terrorism/war | United States | Participants with PTSD^iii^ may be more likely to experience distress from research participation. If participants have indicators of psychological problems, explain how they can connect with services. Informed consent should be clear that participation may cause distress but also be a positive experience. |
| Bowen & Murshid (2016) [46] | Peer reviewed article | Not research | Does not specify | Not specified | Historical/systemic | United States | Focus on training staff who interact with participants to build rapport, ensure confidentiality, educate about the positives of disclosing abuse, ask about abuse, understand domestic violence with an open-lens, elect information with open-ended prompts and well as behaviorally-anchored prompts, avoid stigmatizing questions, and create several chances for disclosure. |
| Brown et al. (2014) [47] | Peer reviewed article | Cross-sectional | Veterans | Adulthood | Terrorism/war | United States | Assess for distress before and after research participation. Research the possibility that trauma research participation contributes to substance use urges and assess potential impact of these urges. |

**Selected Recommendations Pertaining to Safety by Article**

| **Article** | **Publication Type** | **Study Type** | **Population^i^** | **When Trauma Occurred** | **Trauma Type** | **Country** | **Recommendations** |
| --- | --- | --- | --- | --- | --- | --- | --- |
| Campbell et al. (2009) [48] | Peer reviewed article | Qualitative | Women (18-64) who had experienced rape | Adulthood | Sexual | United States | Know that trauma occurs to all demographics and that trauma survivors may express themselves differently: be ready for this diversity when interacting with participants. Know that trauma recovery takes time and different participants will be in different stages of recovery. Recognize that if you have not experienced the trauma that a participant has that you will not be able to completely understand their experience: honor both experiential and academic knowledge. |
| Campbell & Adams (2009) [49] | Peer reviewed article | Qualitative | Women (18-64) who had experienced sexual assault | Adulthood | Physical, sexual | United States | Minimize stigma by having recruitment material highlight empathy and openness. Let participants know that the research may have results that can create positive change, but do not overstate what your study may be able to do. Be explicit about study compensation. |
| Campbell et al. (2010) [50] | Peer reviewed article | Qualitative | Women who had experienced rape | Adulthood | Sexual | United States | Affirm that a participant’s reaction to trauma is normal and provide them with personalized resources for additional support (e.g., point out specific items in a referral list that they may find beneficial.) Communicate warmth and openness. Show empathy when difficult material is disclosed and do not show judgement. Maintain boundaries as a researcher; participants may share very sensitive things but that sharing can only occur in the specific safe container of an interview. Reduce the power difference between researchers and participants by giving participants control over how much they share and inviting participants to ask the researcher questions. |
| Chang et al. (2005) [51] | Peer reviewed article | Qualitative | Women who had experienced intimate partner violence and were accessing services | Adulthood | Intimate partner violence | United States | Create an environment to talk about intimate partner violence by having supportive reading material in the clinic waiting rooms. If a physical exam is required, talk about intimate partner violence before the participant undresses and do not rush the conversation. Let participants know that resources and support and available. |
| Copes et al. (2018) [52] | Peer reviewed article | Qualitative (Photo-voice) | Methamphetamine users in rural Alabama | Not specified | Not specified | United States | Get informed consent to take photos of participants and for participants to send the researchers photos. For consent to be informed, participants must know where the photos may be shared, and that others may recognize their image. |
| Dehghan & Wilson (2018) [53] | Peer reviewed article | Not research | Refugees who have survived sexual torture | Not specified | Sexual | United Kingdom (participants may come from multiple countries though) | Know that not researching refugees’ experiences can be harmful if it keeps their experiences from being acknowledged and addressed. Be aware that potential participants may feel that research participation could help their legal status. Potential participants also may feel research is mandatory if a healthcare professional is connecting potential participants with researchers. |

**Selected Recommendations Pertaining to Safety by Article**

| **Article** | **Publication Type** | **Study Type** | **Population^i^** | **When Trauma Occurred** | **Trauma Type** | **Country** | **Recommendations** |
| --- | --- | --- | --- | --- | --- | --- | --- |
| Douglass et al. (2018) [54] | Peer reviewed article | Not research | Men who experienced partner abuse | Adulthood | Emotional, physical, sexual | Canada, United States, United Kingdom, Australia | Talk with participants about arranging for privacy in advance of data collection if collecting data virtually. Consider participant privacy and confidentiality with respect to what may be visible and who else may be present if using a webcam. Minimize distractions when collecting data online because participants may disclose sensitive information that another participant’s or researcher’s inattention to could be harmful; video may help people be more present. Know that some participants may want to connect with each other after group data collection. Create a process to ask for consent and allow people to be in contact confidentially. |
| Goodwin & Tinderington (2020) [55] | Peer reviewed article | Not research | People who have experienced trauma | Not specified | Not specified | Not specified | Do not exclude trauma survivors from research because it is unjust. Use trauma-informed approaches to research as a norm given how common trauma is. Only ask for necessary information in research. Conduct research in a setting that may feel safe for participants. Describe the steps of the research and how participants’ information will be used. Obtain confidentiality agreements for the data. Offer relevant and trauma-informed referrals to participants as well as those who do not participate. Use CBPR [5]^ii^ methods and recognize systemic and historical trauma. Social work research courses should include information on trauma’s prevalence, trauma-informed approaches to research, and handling vicarious trauma. Training should also be offered on trauma-informed research supervision. |
| Guerra & Pereda (2015) [56] | Peer reviewed article | Mixed methods | Sexually abused adolescents (12-17) in central Chile | Childhood | Developmental/ adverse childhood experiences, sexual | Chile | If working with children, examine the potential effect of research on the child’s mental health. If working with children, collect data individually and do not gather data in a group context. Pilot measures first to see if they may cause distress. |
| Hardesty, Haselschwerdt, & Crossman (2019) [57] | Peer reviewed article | Not research | Individuals involved in qualitative research on interpersonal violence | Adulthood | Vicarious/ secondary | Not applicable | Develop partnerships with communities before initiating any research. Make sure your staff are well-trained in safety and ethics. Allow potential participants to initiate contact if interested in research during recruitment. Design recruitment materials with the assumption that an abuser may see the materials: do not advertise that the study is on violence. Practice reflexivity. Have protocols and referrals for participant distress and provide referrals to all participants at the end of the interview. These referrals should include resources for domestic violence nested within referrals to other resources. End interviews with empowering questions. |
| Jorm, Kelly, & Morgan (2007) [58] | Peer reviewed article | Review | Individuals with symptoms of or risk factors for a psychiatric disorder | Childhood and adulthood | Multiple | Not applicable | Coordinate with other researchers to avoid over-researching a population. Informed consent should be appropriate to the cognitive/mental capacity of participants and should say the risks but also that participation can be positive. Consider providing example difficult questions in the consent process. Ensure participants are in a place where they can give unimpaired consent and give information in writing. Develop a plan to support distressed participants. Don’t collect data immediate after the trauma was experienced. Let participants decide when and where to interview, how long to go, and if multiple sessions should be used. Be ready to check in with distressed participants during data collection and be ready to stop an interview if needed. Have appropriate referrals on-hand. De-brief after interviewing. |

**Selected Recommendations Pertaining to Safety by Article**

| **Article** | **Publication Type** | **Study Type** | **Population^i^** | **When Trauma Occurred** | **Trauma Type** | **Country** | **Recommendations** |
| --- | --- | --- | --- | --- | --- | --- | --- |
| Kyegombe et al. (2019) [59] | Peer reviewed article | Not research | Children with disabilities | Childhood | Developmental trauma/ adverse childhood experiences (ACEs) | Not applicable | Consider if it is appropriate to seek consent from a parent or organization that can act in a guardianship capacity if a participant’s ability to give informed consent may be lacking. However, seek input from the potential participant to allow for supported decision-making rather than substituting their decision-making. Also, assess participants’ capacities rather than relying on caregiver reports of capacity, ensure participation is accessible for children with disabilities, be wary about potential coercion if data are collected in school settings where opting out may be dissuaded, and use CBPR [5]^ii^ methods. |
| Kimberg (2008) [60] | Peer reviewed article | Not research | Men who were victims or perpetrators of intimate partner violence | Childhood and adulthood | Intimate partner violence | United States | Assess the participant’s potential past and current role in abuse. Evaluate current safety considering threats of murder to or from the participant, availability of weapons, history of violence or stalking, suicidality, and risks to children. Assess effects of abuse. Evaluate participants’ stages of change and their capacity for change. Repeatedly tell survivors that they are not to blame for the abuse. Repeatedly tell perpetrators that the abuse and stopping it is their responsibility. Repeatedly say to everyone that abuse is bad and hurts relationships and families. Make hotline numbers available. Discuss and develop safety plans and plans to stop abuse, including connecting a perpetrator with services. If necessary, encourage a participant to access or access an involuntarily psychiatric hold and notify whoever may be in danger as well as police. Offer referrals to police and legal aid. Plan follow ups with participants and ensure you have a safe way to connect with them. |
| Linabary & Corple (2019) [61] | Peer reviewed article | Not research | Individuals participating in online research | Adulthood | Emotional trauma | Not applicable | When working with online data, be aware that it may not be clear what is public versus private information. Know that participants may have different privacy vulnerabilities in research due to their positionality with respect to privilege and marginalization. Consider how participants’ and your own social locations affect vulnerability in research and ways to minimize it, how participants may view privacy, and ways to engage with and be accountable to those who may be most negatively affected by privacy violations. |
| Mwambari (2019) [62] | Peer reviewed article | Qualitative | Individuals in Northern Uganda who have worked with research | Not specified | Not specified | Northern Uganda | Outsider researchers frequently create risk for local research assistants and community members by not properly attending to issues of power, safety, or positionality. Training may help researchers attend to these issues. |

**Selected Recommendations Pertaining to Safety by Article**

| **Article** | **Publication Type** | **Study Type** | **Population^i^** | **When Trauma Occurred** | **Trauma Type** | **Country** | **Recommendations** |
| --- | --- | --- | --- | --- | --- | --- | --- |
| Pickles (2020) [63] | Peer reviewed article | Not research | Individuals 13+ involved in LGBT^iv^ organizations in North East England | Childhood and adulthood | Hate crimes | United Kingdom | Requiring parental consent for sexual minority minors to participate in research can be harmful, and if IRBs^[[6]](#endnote-6)^ question minors’ knowledge of their sexuality they can perpetuate homophobia. Rather, risk should be minimized for minors and safeguards for them to participate may be put in place. For example, non-parental adults can provide in loco parentis consent in some cases and minors should still be asked for informed assent. Minors should also be asked about their understanding of the research project as part of an informed assent process. |
| Rivlin et al. (2012) [64] | Peer reviewed article | Cross-sectional, case-control | Prisoners in the United States, young offenders, adults. | Adulthood | Emotional | United States | Ensure participants know the nature of the interview and what topics may be covered in order to minimize the possibility of distress. Assess feelings with a short but valid measure before and after data collection to see if a participant may need additional support to process the interview experience. Consider having a different researcher or an anonymous questionnaire to assess reactions to research. In particular, participants in prison may feel pressure to participate because they are in an environment where they are constrained. |
| Scerri, Abela, & Vetere (2012) [65] | Peer reviewed article | Qualitative | Adult women who had experienced domestic violence during childhood | Childhood | Emotional, physical | United Kingdom | Distinguish between responsibilities of clinicians and researchers. Make sure participants have clinical support and they have the opportunity for reflection with an appropriate mental health provider if needed. De-brief with participants after data collection and give participants multiple opportunities to give or refuse consent. |
| Schwerdtfeger (2009) [66] | Peer reviewed article | Qualitative, Cross-sectional | Second or third trimester pregnant women in the Southwest United States | Adulthood | Sexual | United States | Develop rapport before asking about sexual trauma. To avoid stigmatization, pay attention to the language participants use to describe their trauma and use that language. Be sure to pace questions to account for emotional burden. If there will be follow-up say how it may be different from earlier data collection. Be upfront that the research interview is not designed to be therapeutic. |
| Sullivan and Cain (2004) [67] | Peer reviewed article | Not research | Not applicable | Not applicable | Intimate partner violence, sexual, physical, emotional | United States | Enact safety measures to decrease risk of abuse that participants may experience if an abuser learns of their research participation. These should be present throughout protocols for recruitment, data collection, location, and participant tracking in longitudinal research. |
| Testa, Livingston, & VanZile-Tamsen (2011) [68] | Peer reviewed article | Not research | Not applicable | Childhood and adulthood | Sexual | Not specified | If a participant is distressed during data collection be ready to stop or take a break. Consider using written narratives to supplement or replace other data collection modes. |

**Selected Recommendations Pertaining to Safety by Article**

| **Article** | **Publication Type** | **Study Type** | **Population^i^** | **When Trauma Occurred** | **Trauma Type** | **Country** | **Recommendations** |
| --- | --- | --- | --- | --- | --- | --- | --- |
| Waechter et al. (2019) [69] | Peer reviewed article | Cross sectional | Youth involved with child protective services | Childhood | Developmental/ adverse childhood experiences | Canada | Know that participants with trauma-related mental illness may find participating in research more difficult than participants without trauma-related mental illness, but they may also find it more rewarding. When conducting research with adolescents who have a trauma background emphasize that participants have a right to withdraw and retract their data at any time. Be forthright about any confidentiality limits, carefully monitor for potential participant distress during and after participation, and only ask potentially invasive questions that may be necessary. |

**Selected Recommendations Pertaining to Researcher Well-Being by Article**

| **Article** | **Publication Type** | **Study Type** | **Population^i^** | **When Trauma Occurred** | **Trauma Type** | **Country** | **Recommendations** |
| --- | --- | --- | --- | --- | --- | --- | --- |
| Adams (2020) [70] | Dissertation | Qualitative | Male mental health professionals within the United States | Adulthood | Emotional (compassion fatigue) | United States | To manage compassion fatigue use social support, knowledge, staying connected to clients, reducing stress, and fostering compassion satisfaction. |
| Berger (2020) [71] | Peer reviewed article | Narrative review | Trauma researchers | Adulthood | Vicarious/ secondary | Not specified | Advocate for awareness of and support for handling research-related trauma within institutions. Research-related trauma is common, IRBs^vi^ should require that trauma potential for researchers be addressed, and training on research-related trauma should be provided. Researchers also need to practice self-care and institutions should support researcher examination of how trauma affects them. |
| Berger & Quiros (2016) [72] | Peer reviewed article | Qualitative | Supervisors of trauma-informed social work practices | Not specified | Multiple | United States | Suggestions pertained to supervision for trauma-informed practice but may apply to supervision for trauma-informed research: Empower supervisees. Focus on relational interaction and educational growth; discuss countertransference that may arise when working with people with trauma and provide support while also maintaining boundaries as a supervisor. Bring up and make space for supervisees to explore their own trauma reactions and self-care. Model effective responses to trauma and self-care. Emphasize the importance of safety and taking care of oneself. |
| Btoush & Campbell (2009) [73] | Peer reviewed article | Not research | Not applicable | Adulthood | Intimate partner violence | United States | Provide researchers with regular debriefing, discuss trauma openly, and plan to address secondary traumatization and researchers’ own experiences with trauma. Allow researchers to stop work on a project without repercussions. |

**Selected Recommendations Pertaining to Researcher Well-Being by Article**

| **Article** | **Publication Type** | **Study Type** | **Population^i^** | **When Trauma Occurred** | **Trauma Type** | **Country** | **Recommendations** |
| --- | --- | --- | --- | --- | --- | --- | --- |
| Coles & Mudaly (2010) [74] | Peer reviewed article | Not research | Researchers who conduct interviews with children who have experienced sexual abuse and young mothers who were sexually abused in childhood. | Adulthood | Emotional | United States | Ensure that supervisors can respond to potential traumatization among staff. During training, anticipate and describe potential impacts of the study on staff. Discuss addressing researcher safety in ethics applications. Researchers should prepare family and friends for the possible impact that the research may have on them. Schedule supervision, opportunities for peer support, and de-briefing regularly. Give access to crisis counseling. Strive to be consciously empathetic when collecting data. Break up intense interviews into multiple sessions. Keep a reflective journal of thoughts and emotions during data collection. Make sure workloads are manageable. Cap your involvement with research data (e.g., no more than one interview in a day). Disseminating research may help process emotional impact; consider multiple formats, dissemination with communities, and using to take political action or advocate for policy change. |
| Coles et al. (2014) [75] | Peer reviewed article | Qualitative | Sexual violence researchers | Adulthood | Emotional | Australia | Understand distinction between research and therapy and have appropriate referrals in place for participants. Researchers should emotionally prepare themselves for trauma research. In particular, there should be no career consequences if early career researchers opt out of conducting trauma research. |
| Eadesa et al. (2020) [76] | Peer reviewed article | Qualitative | Aboriginal and Torres Island Strait women managing chronic disease | Childhood and adulthood | Developmental/ adverse childhood experiences (ACEs), emotional, physical, vicarious/ secondary, historic/systemic | Australia | Consider the safety of researchers as well as participants, especially when researchers may experience systemic trauma themselves. Discuss vicarious trauma and have trauma-informed supervision, debriefing, and access to counselling. |

**Selected Recommendations Pertaining to Researcher Well-Being by Article**

| **Article** | **Publication Type** | **Study Type** | **Population^i^** | **When Trauma Occurred** | **Trauma Type** | **Country** | **Recommendations** |
| --- | --- | --- | --- | --- | --- | --- | --- |
| Fohring (2020) [77] | Peer reviewed article | Not research | Qualitative researchers working on difficult topics | Adulthood | Emotional, vicarious/ secondary | Not specified | Expect trauma research to require emotional labor, generate overwhelming feelings, and that you may feel bad for having adverse reactions to your research. Be aware that you may also be frustrated that as a researcher you are not directly helping participants but are instead identifying larger patterns, and that this may or may not help participants. Know that secondary traumatic stress and vicarious trauma may both occur; however, post traumatic growth is possible. You can try to offset the negative feelings associated with your research through engagement, re-examining beliefs, reflexivity, and gathering resources. Seek out counselling, trauma-informed supervision and training, and social support. Also, access forums, resources, and trainings that may be helpful such as those available through Pearlman’s [Headington Institute](https://www.headington-institute.org/), [78] and the [Sexual Violence Research Initiative](http://www.svri.org) [79]. Research methods course instructors need to include researcher well-being in their instruction. Research institutions, publishers, and funders need to collectively address researcher well-being |
| Guerzoni (2020) [80] | Peer reviewed article | Not research | Researchers/academics/students who may work on trauma | Adulthood | Vicarious trauma from research on child sex abuse | Not specified | Anticipate that researching trauma will cause secondary trauma. Universities and trauma researchers should teach students about secondary trauma and should provide structure to address it (e.g., required counselling.) |
| Jeftic (2020) [81] | Peer reviewed article | Qualitative | People who survived the war in Sarajevo | Not specified | Terrorism/War, vicarious | Bosnia and Herzegovina | Utilizing empathy and perspective-taking may help when hearing difficult narratives during research, but it is not socially acceptable to discuss what you hear without showing emotion and condemning abuse. This can create a dilemma and make you distance yourself from trauma research. It may help if you know about other research projects on the same theme. Build support networks with other researchers and address vicarious trauma. |

**Selected Recommendations Pertaining to Researcher Well-Being by Article**

| **Article** | **Publication Type** | **Study Type** | **Population^i^** | **When Trauma Occurred** | **Trauma Type** | **Country** | **Recommendations** |
| --- | --- | --- | --- | --- | --- | --- | --- |
| Markowitz (2021) [82] | Peer reviewed article | Not research | Trauma researchers/academics | Adulthood | Emotional | El Salvador | Accept that talking about researcher emotion is important to the research process and for the well-being of researchers. Recognize barriers to talking about emotions connected to doing research: positivism is dominant, and you may fear potential costs of talking about emotion or incompletely understand positionality. Expect that talking with perpetrators of violence will invoke strong feelings and talking with survivors may invoke helplessness. Know that you may face additional binds in that ethics boards may require researchers to secure permissions from gatekeepers, which could make research participation unsafe for potential collaborators or participants and may prevent researchers from coming into the research setting as a neutral party. An additional weight is that while researchers may fear distressing participants with questions, not asking participants about painful experiences may be tantamount to erasure. Because research invokes such powerful emotions, talking about researcher emotion needs to be normalized and ethics boards should consider researcher well-being as well as participant safety. Researchers may benefit from having a sense of what they may experience before beginning a research project, knowledge of coping techniques, introspection to detect how one is doing, knowledge of where to access support, trauma-informed supervision and buddy systems, and recognition of their work. |
| Nikischer (2019) [83] | Peer reviewed article | Qualitative | Trauma researchers/academics | Adulthood | Emotional | Not specified | The potential for vicarious traumatization needs to be addressed in IRB^vi^ applications, research training programs, and in the work responsibility expectations of academics working on trauma. |

**Selected Recommendations Pertaining to Researcher Well-Being by Article**

| **Article** | **Publication Type** | **Study Type** | **Population^i^** | **When Trauma Occurred** | **Trauma Type** | **Country** | **Recommendations** |
| --- | --- | --- | --- | --- | --- | --- | --- |
| Smith et al. (2019) [84] | Peer reviewed article | Qualitative | Novice trauma researcher interviewers | Adulthood | Emotional, vicarious/ secondary | United States | Be aware that novice trauma research interviewers may experience resilience and post-traumatic growth, but also secondary traumatic stress, vicarious trauma, and compassion fatigue. Training should cover these constructs, how interviewers may experience them, countertransference, the importance of de-briefing, de-briefing techniques, self-care, and seeking support. Training should also emphasize that there are many ways to process an interview, that interviewers’ reactions may change over time, and that interviewers may have contradictory reactions to the interviews. Utilize trauma-informed supervision that emphasizes self-care and regular de-briefings. Know that participants in trauma research often do not regret their research involvement and may report benefits from participating in research. |
| Starcher & Stolzenberg (2020) [85] | Peer reviewed article | Cross-sectional | Forensic interviewers working at child advocacy centers | Adulthood | Vicarious/ secondary | United States | Know that conducting forensic interviews may place you at risk for PTSD^iii^ and burnout: heavy workload and decreased support associates with burnout whereas increased support is protective against it. Interviewers should have trauma-informed supervision, debriefing opportunities, ongoing training, and access to mental health services. Because the combination of trauma from conducting interviews and racism may place interviewers of color at increased risk of burnout or PTSD, organizations should address racism within and seek to promote support between workers. |
| Taylor et al. (2016) [86] | Peer reviewed article | Review | Papers reporting primary empirical research in trauma between January 1, 2009 and December 31, 2014. | Childhood and adulthood | Emotional, vicarious/ secondary | United Kingdom | The risk of vicarious trauma to researchers is minimal when working with non-primary data on non-vulnerable topics. The risk is medium if working with non-primary data on vulnerable topics or primary data on non-vulnerable topics. The risk is highest when working with primary data on vulnerable topics. |

**Selected Recommendations Pertaining to Researcher Well-Being by Article**

| **Article** | **Publication Type** | **Study Type** | **Population^i^** | **When Trauma Occurred** | **Trauma Type** | **Country** | **Recommendations** |
| --- | --- | --- | --- | --- | --- | --- | --- |
| Tsai et al. (2018) [87] | Peer reviewed article | Qualitative | Adults (18+) in Cebu City, Philippines who experienced sex trafficking and their family members | Adulthood | Sexual | Philippines | Plan for vicarious traumatization- discuss what trauma research is like and the risk of vicarious trauma with researchers as part of training. Allow funding and time for handling vicarious traumatization on the research team and provide a venue for researchers to process their experiences safely.  Anticipate likely scenarios that may arise before collecting data and train all staff on response plans, including pre-screened resources for referrals. |
| Wager (2011) [88] | Peer reviewed article | Mixed methods, cross-sectional survey and prospective follow-up study | Adults who had experienced childhood sexual abuse | Childhood | Sexual | United Kingdom | Trauma research can have researcher costs. Plan for your research to be personally challenging; seek input from others in light of this and modify your protocols accordingly before you start your study. Researchers may experience vicarious trauma, or they may have personal trauma histories themselves. Researchers may feel like they don’t have control and that they are not helping a participant due to the limited nature of research interactions. Researchers with a trauma history may be particularly sensitive to this. Conducting trauma research may cost the researcher and others in their network; the researcher should plan for and prioritize self-care. Self-care requires balance, social connection, and self-awareness. Researchers should also know that they may learn something from participants that challenges how they see the world or their own trauma experience, which can be very disorienting. In light of this, researchers should anticipate needing time to procrastinate and time to reflect on what they are learning. Researchers with a trauma history should know though that they have an invaluable role in trauma research: they may dig deeper into a topic and they may be more likely to use study results to create change. They may also benefit from conducting trauma research through experiencing post-traumatic growth. |

**Selected Recommendations Pertaining to the Nature and Scope of Trauma Research by Article**

| **Article** | **Publication Type** | **Study Type** | **Population^i^** | **When Trauma Occurred** | **Trauma Type** | **Country** | **Recommendations** |
| --- | --- | --- | --- | --- | --- | --- | --- |
| Abu-Rus et al. (2019) [89] | Peer reviewed article | Qualitative | Participants in trauma research | Not specified | Not specified | United States | Be aware that informed consent to participate in trauma research could have a nocebo effect and could discourage participants from disclosing trauma, exacerbating trauma avoidance symptoms. Professional organizations that work on trauma and research should develop recommendations for discussing differing types of risk in informed consent processes. |
| Acierno et al. (2003) [90] | Peer reviewed article | Cross-sectional | Adults (55-85) residing in the Southeastern United States | Childhood and adulthood | Emotional, physical, sexual | United States | Inform participants what types of trauma are relevant (e.g., any assault experienced any time in life). Don’t ask about assault after a psychopathology survey. Use behaviorally specific, closed-ended questions to minimize variance and ease participant burden. |
| Alaggia, Collin-Vezina, & Lateef (2019) [91] | Peer reviewed article | Review | Survivors of childhood sexual abuse of all ages | Childhood | Sexual | Not indicated | Research disclosure of childhood sexual abuse across different life stages, especially young and later adulthood. |
| Baumgartner et al. (2015) [92] | Peer reviewed article | Mixed methods | Adolescent (15-21) women in Tanzania | Childhood | Physical | Tanzania | When screening for abuse, ask questions about what specific experiences participants have had (e.g., ever been hit) rather than asking them to label experiences as abuse or trauma or asking them to connect their experiences with emotions such as fear. |
| Berg & Mulford (2020) [93] | Peer reviewed article | Review | Publications on the victim-offender overlap | Not specified | Victimization | Not specified | Design studies that account for participants being both victims and offenders. Know that current time lag approaches used in research may be ill-specified and that qualitative and mixed methods research may provide valuable information about the environments and contexts in which victimization and offending occurs. |
| Binion & Gray (2020) [94] | Peer reviewed article | Review | LGB^iv^ people 14 or older who have experienced single incident sexual assault | Childhood and adulthood | Sexual | United States | Conduct qualitative research to identify cognitive distortions unique to sexual minorities who have experienced sexual assault, longitudinal research, and research on perpetrators. Incorporate intersectionality (Crenshaw, 1989) [9] into your work. |
| Blasbalg et al. (2019) [95] | Pre-print of accepted article | Mixed methods | Children being interviewed in abuse investigations | Childhood | Developmental/ adverse childhood experiences (ACEs), physical | Israel | Get trained in supportive interviewing techniques so as to better interview children who may be reluctant to disclose abuse. |
| Boals, Contractor, & Blumenthal (2020) [96] | Peer reviewed article | Review | College students and general population | Childhood and adulthood | Any trauma | Not specified | Know that college samples may be representative samples for trauma research. They have diverse trauma exposures, symptoms, and correlates of trauma. Be aware that college samples may be beneficial because large samples can be obtained, sub-group analyses are possible, and prospective research is feasible. |

**Selected Recommendations Pertaining to the Nature and Scope of Trauma Research by Article**

| **Article** | **Publication Type** | **Study Type** | **Population^i^** | **When Trauma Occurred** | **Trauma Type** | **Country** | **Recommendations** |
| --- | --- | --- | --- | --- | --- | --- | --- |
| Bouchard (2016) [97] | Opinion | Not research | Youth | Childhood | Emotional | Canada | Know that online research may be a novel way to characterize the phenomenology of experiencing trauma. By providing participants both anonymity and a group setting, you can provide a safe, supportive opportunity to build rapport with others and share their experiences. It may help you build rapport with participants as well. However, there are questions of validity and logistics to consider, as detailed in the article. |
| Carr (2019) [98] | Peer reviewed article | Not research | Researchers who are also mental health service users | Adulthood | Emotional | United Kingdom | Know that patient and public involvement may not currently be successful in giving mental health service users and survivors a role in knowledge production. Be aware that much remains unknown about what patient and public involvement in research looks like, how much power and influence patients have in this research, characteristics of patients and members of the public who are involved in this research, successful research outcomes, or practical and ethical challenges in this research. Researchers who are also mental health service users may experience marginalization in academia, a greater emotional work toll than researchers who do not live with mental health challenges, little tailored occupational support, and ‘othering’ from other mental health service users. Some researchers who are mental health service users seek to affect change through their research, or by providing bridging social capital between knowledge brokers and wider communities of mental health service users. Accomplishing this requires connection though, or bonding social capital, with mental health service users outside academia. Situated solidarity is a frame that researchers who are mental health service users may find useful. |
| Cattaneo & Chapman (2011) [99] | Peer reviewed article | Qualitative | Intimate partner violence service providers | Not applicable | Intimate partner violence | Not specified | Know that the choice of screening methodology for intimate partner violence has implication for provider and patient comfort. If service referrals will be the same for all, consider using a brief screening rather than in-depth assessment. Assess how well these instruments operate by looking at services that participants receive. Study what positive impacts participants may gain from risk assessments and after data collection ask them what they saw as the potential negatives of the risk assessment. |
| Cederborg & Lamb (2008) [100] | Peer reviewed article | Observational | People with intellectual disabilities reporting a crime | Childhood and adulthood | Not specified | Sweden | Open-ended questions should be used in research among trauma survivors with intellectual disabilities. |

**Selected Recommendations Pertaining to the Nature and Scope of Trauma Research by Article**

| **Article** | **Publication Type** | **Study Type** | **Population^i^** | **When Trauma Occurred** | **Trauma Type** | **Country** | **Recommendations** |
| --- | --- | --- | --- | --- | --- | --- | --- |
| Chae et al. (2011) [101] | Peer reviewed article | Not research | Children who experienced or witnessed trauma | Childhood | Developmental | United States | Understand that partnering with service organizations or legal agencies for recruitment can be beneficial but each comes with a set of challenges and costs. If you have a control group of people without trauma be cautious of the fact that screening participants for a control group may uncover trauma experiences and mandating reporting requirements may apply; try to minimize this happening by stating the criteria for control participants and asking that people who have had experiences of trauma not try to sign up as control participants. |
| Chan, Teram, & Shaw (2017) [102] | Non-peer reviewed article | Not research | Adults and children who experienced childhood sexual abuse | Childhood | Sexual | China, United States | Ensure that the research design reflect participants’ preferences. |
| Chozinski & Gonzalez (2020) [103] | Peer reviewed article | Qualitative/Photo-voice | Undergraduates at a Catholic and Hispanic-serving university | Not indicated | Not specified | United States | Consider that photo voice may make research participation accessible to more people. Photos can also be geotagged. |
| Clark et al. (2012) [104] | Peer reviewed article | Mixed methods | Women in Amman, Jordan who experienced domestic violence | Childhood and adulthood | Developmental, physical, sexual | Jordan | Know that many participants will have a positive reaction to research and even if they have a negative reaction, they may still find the research to be important. There needs to be research to understand participant reactions. |
| Clark & Walker (2011) [105] | Opinion, commentary on article | Not research | Survivors of trauma | Childhood and adulthood | Not specified | Not applicable | Research training should include and highlight how to formulate and test empirical hypotheses about ethics. |
| Collings (2019) [106] | Peer reviewed article | Cross-sectional | Social science undergraduate students | Childhood and adulthood | Intimate partner violence (emotional, physical, sexual) | South Africa | Know that participants who rate their participation in trauma research as minimal risk will report positive benefit to risk ratios. Factors that increase a participant’s perceived risk in research include emotional abuse, PTSD,^iii^ depression, and older age. |

**Selected Recommendations Pertaining to the Nature and Scope of Trauma Research by Article**

| **Article** | **Publication Type** | **Study Type** | **Population^i^** | **When Trauma Occurred** | **Trauma Type** | **Country** | **Recommendations** |
| --- | --- | --- | --- | --- | --- | --- | --- |
| Decker et al. (2011)[107] | Peer reviewed article | Qualitative, Cross-sectional | Female undergraduates (18-44) | Childhood | Developmental/ adverse childhood experiences (ACEs) | United States | Study the research experience for participants with validated tools such as the Reactions to Research Participation Questionnaire.[108] Research benefit and not just distress. It may be helpful for participants to disclose abuse experiences to an empathetic listener and this should be studied. Use findings to help shape future study design to support beneficial experiences. Train researchers on how to investigate the research experience for participants and how to further research on this topic. Train IRBs^vi^ on this topic too and support efforts to enhance IRBs making decisions using empirical data. Let IRBs know that participants may experience benefit from participation, that harm may be less likely than anticipated, and that there may not be long-term distress experienced from participating in trauma research. |
| Danese (2020) [109] | Peer reviewed article | Not research | Individuals with childhood trauma | Childhood | Developmental/ adverse childhood experiences (ACEs) | Not specified | Do not use prospective and retrospective measures of childhood trauma interchangeably as they may capture different concepts. Do not assume that children with trauma only differ from children without trauma on the basis of trauma. They may have pre-existing vulnerabilities. Consider machine learning methods to identify risks of harm from trauma. |
| Dell et al. (2019) [110] | Peer reviewed article | Review | Individuals who had been trafficked who participated in an intervention to support trafficking survivors | Not specified | Physical, sexual | Not specified | Research whether continuum of care interventions or simpler targeted interventions are more beneficial. Researchers can collaborate on studies and to advocate for funding, and need to focus on improving the quality of studies. Research mediators and moderators and define core outcome sets with input from researchers and survivors. |
| DePrince & Chu (2008) [111] | Peer reviewed article | Cross-sectional, Experimental | Residents of a large city in the United States; Undergraduates | Childhood and adulthood | Developmental/ adverse childhood experiences (ACEs), emotional, physical, sexual, natural disaster, historical/systemic, terrorism/war | United States | Collect the Reactions to Research Participation Questionnaire [108] or otherwise assess participant experiences (for sample questions see DePrince & Freyd, 2006).[112] Information is needed on how method and person-level factors such as demographics and trauma exposure affects participant experiences. Collecting data on participant experiences with research may allow for systematic analyses and that can lead to trauma research improvement. Include data on participant experiences, including perceived risks and benefits, in your IRB^vi^ renewals; these data can educate IRBs. |

**Selected Recommendations Pertaining to the Nature and Scope of Trauma Research by Article**

| **Article** | **Publication Type** | **Study Type** | **Population^i^** | **When Trauma Occurred** | **Trauma Type** | **Country** | **Recommendations** |
| --- | --- | --- | --- | --- | --- | --- | --- |
| Draucker, Martsolf, & Poole (2009) [113] | Editorial | Not research | Children and adults who experienced sexual abuse | Childhood and adulthood | Sexual | United States | Consider the inhibition-confrontation model as it applies to research experiences, and factors that could influence benefits and harms of research participation. Collect qualitative data and collect data that is systematic on what research participation was like for participants, both during and after the study. A reflective journal may help keep track of observations. Know that non-data collection research tasks could evoke emotion. |
| Finkelhor et al. (2014) [114] | Peer reviewed article | Cross-sectional | Children (10-17) in the United States | Childhood | Developmental/ adverse childhood experiences (ACEs) | United States | Know that trauma research may cause distress but not as often as commonly believed. Even if research participation is upsetting, participants generally say that they would partake in the study again. To address this misunderstanding, IRBs^vi^ should consider requiring that researchers collect data on negative research experiences or conducting systematic reviews evaluating study methods with respect to subsequent participant distress. |
| Gagnon et al. (2015) [115] | Peer reviewed article | Experimental, convenience sample. | Adults (60+) | Not specified | Developmental, emotional, physical, sexual | United States | Know that many participants have positive experiences with research and often positive experiences outweigh negative experiences; researchers should design studies to have more positive experiences for participants. Elders should not be screened out of research participation if they have the ability to provide consent. Depression and trauma are associated with having negative experiences with research. |
| Gaillard & Peek (2019) [116] | Peer reviewed article | Not research | Residents who have lived through a disaster | Not specified | Disaster | Applicable to all countries | Recognize that local researchers and outsider researchers may have different agendas. Outsider researchers may be positioned to better take advantage of resources to pursue their agenda. Local researchers may approach outsider researchers to help on projects and not have their contributions appropriately acknowledged or utilized. There needs to be a directory of local researchers when disaster occurs and there needs to be mechanisms to ensure local input and control into disaster research. |

**Selected Recommendations Pertaining to the Nature and Scope of Trauma Research by Article**

| **Article** | **Publication Type** | **Study Type** | **Population^i^** | **When Trauma Occurred** | **Trauma Type** | **Country** | **Recommendations** |
| --- | --- | --- | --- | --- | --- | --- | --- |
| Galea et al. (2005) [117] | Peer reviewed article | Experimental | New York City residents | Not specified | Terrorism/war | United States | Know that many participants do not find participation in trauma research distressing, but it is helpful to be able to connect those who do with services. Participants are more likely to find research distressing if they are middle aged, women, unmarried, lacking access to health care, directly affected by trauma, or have depression or other mental health issues. |
| Golden (2019) [118] | Dissertation | Mixed methods | Girls and women 11-21 in Louisville | Childhood | Violence | United States | Do not rely on direct communication, consider alternative forms of data collection such as creative writing, allow open ended answers, and accommodate expanded definitions of constructs. Education of researchers should focus on responsivity to culture and trauma, innovation, creative methodologies, and understanding power and oppression critically in order to support needed methodological changes. Researchers also need to understand that their choice of methodologies may directly benefit participants. |
| Gonçalves & Matos (2020) [119] | Peer reviewed article | Cross-sectional | Immigrant women in Portugal | Adulthood | Emotional, physical, sexual, terrorism/war | Portugal | Consider that much of the violence that immigrant women in Portugal face occurs outside the home and is not domestic violence. Researchers need to be aware of this to accurately measure violence. |
| Goodkind et al. (2017) [120] | Peer reviewed article | Mixed methods | Adult refugees from Afghanistan, the Great Lakes Region of Africa, and Iraq | Not specified | Not specified | United States | Evaluate interventions using mixed-methods. Document intervention diffusion and community change, and assess participant perceptions of the impact of community change on their lives. Note participants’ positive comments about participating. Randomized control trials may test participant and community trust. Before accepting participants, have separate group orientations for each demographic group where you explain your research design and your qualified commitment to the community. When working with community members on the research team, allow for reflection, be willing to adapt study protocols, be open to learning from them, and offer substantial straining and support. Offer evidence-based care to control participants who are negatively affected by PTSD.^iii^ |
| Gultekin et al. (2019) [121] | Peer reviewed article | Not research | Not specified | Not specified | Not specified | Not specified | Researchers should use a framework for approaching the impacts of trauma that addresses components of intersectionality (Crenshaw, 1989) [9], development, and life attainment. Targets for intervention are identity, self-regulation, relationships, and safety. |

**Selected Recommendations Pertaining to the Nature and Scope of Trauma Research by Article**

| **Article** | **Publication Type** | **Study Type** | **Population^i^** | **When Trauma Occurred** | **Trauma Type** | **Country** | **Recommendations** |
| --- | --- | --- | --- | --- | --- | --- | --- |
| Hoover & Marrow (2015) [122] | Organizational report | Qualitative | Women (23-24) | Adulthood | Emotional, physical, sexual | United States | Investigate participant experiences with research and practice reflexivity to improve your research. Research is needed examining long-term impacts of and perspectives toward research participation, dynamics between researchers and participants, and dynamics between participants. Additionally, investigating researcher reflexivity may create helpful knowledge on researcher development. |
| Keyes, Vogel-Ferguson, & Patin (2019) [123] | Peer reviewed article | Not research | Rural predominately Native American and white community | Not specified | Historic/systemic | United States | Recognize that CBPR [5] ^ii^ and social work share several values: recognizing that the practitioner’s primary responsibility is to those that their work purports to benefit (e.g., community or client) and having them led the focus of the work, and valuing self-determination, collaboration, and relationships. Both value reflexivity and addressing power dynamics within society as well as the work being done by the practitioner. CBPR [5] is a valuable tool. |
| Kim & Schmuhl (2019) [124] | Peer reviewed article | Review | LGBTQ+^iv^ | Adulthood | Intimate partner violence | United States | If you are working on intimate partner violence among LGBTQ+^iv^ populations try to publish in broader violence journals and not just LGBT focused ones. Because so little intimate partner violence research focuses on LGBT populations, there may need to be more theoretical development that researchers can use. Conduct inter-disciplinary research and use validated measures. It may be necessary to develop LGBT focused measures. |
| Massey (2013) [125] | Peer reviewed article | Cohort | Adults | Childhood | Developmental/ adverse childhood experiences (ACEs) | United States | Research participant experiences with research with measures such as the Reactions to Research Participation Questionnaire [108]. This is important as trauma research proliferates. Disseminate your finding so that we can learn what characteristics may contribute to having a bad experience participating in research. |
| Matheson & Weightman (2019) [126] | Peer reviewed article | Qualitative | Patient-researchers with complex post-traumatic stress disorder | Not specified | Not specified | United Kingdom | Attend to power dynamics in work with patient-researchers. Also, know that patient-researchers may see their participation as empowering. |

**Selected Recommendations Pertaining to the Nature and Scope of Trauma Research by Article**

| **Article** | **Publication Type** | **Study Type** | **Population^i^** | **When Trauma Occurred** | **Trauma Type** | **Country** | **Recommendations** |
| --- | --- | --- | --- | --- | --- | --- | --- |
| Newman & Kaloupek (2004) [127] | Peer reviewed article | Mixed methods | Not applicable | Childhood and adulthood | Emotional, physical, sexual historical/systemic | United States | Know that trauma does not preempt someone from being able to give informed consent. Screening someone out of research participation on the basis of a trauma history violates the principle of autonomy. Evidence indicates that participants may feel distress recalling their past negative experiences, but few regret research participation. Distress may actually indicate engagement rather than harm. Anticipate potential ethics issues or issues related to participant safety. Write each potential issue as a hypothesis and review the literature and talk with researchers experienced on your study topic. Use this to inform the research protocol and, following the study, collect information on how the protocol worked for participants. Use data collected in your study on participant experiences, distress, benefits, and harms, to adapt your protocol as needed. Do not refer to research as having a potential to re-traumatize: this equates recollection with the actual trauma experienced and this erases that the nature of trauma is an experience of intense threat that one has no control over. In research, efforts are made to give participants control so use of the word re-traumatizing creates an unnecessarily high focus on research risks and prevents a balanced view of risks and benefits from being considered. Key gaps for the field of reactions to trauma research include who is most likely to experience distress (or benefit), how participant benefit long-term, how distress differs from typical symptoms, and how research distress occurs in other topics. |
| O’Brien (2019) [128] | Peer reviewed article | Review | Youth participating in bullying research | Childhood | Bullying, developmental/ adverse childhood experiences (ACEs) | Not specified | Conduct participatory research with youth on issues affecting youth because youth may understand situations differently than adults. Partnering with youth in research on issues that affect youth will thus help researchers understand their topic of inquiry better. |
| Øverlien, Hellevik, & Korkmax (2020) [129] | Peer reviewed article | Qualitative | Young women who have experienced intimate partner violence within a heterosexual relationship | Childhood and adulthood | Intimate partner violence | Sweden and Norway | To do in-depth research on violence in relationships, investigate characteristics of relationships, and the context and situation that violence occurs in. |

**Selected Recommendations Pertaining to the Nature and Scope of Trauma Research by Article**

| **Article** | **Publication Type** | **Study Type** | **Population** | **When Trauma Occurred** | **Trauma Type** | **Country** | **Recommendations** |
| --- | --- | --- | --- | --- | --- | --- | --- |
| Pickering & Maxwell (2018) [130] | Peer reviewed article | Cross-sectional | Seniors in the United States identified as experiencing abuse through the police | Adulthood | Senior abuse and neglect | United States | Know that partnering with police to identify seniors who have experienced abuse and neglect from police incident reports may be feasible. |
| Rosenstein (2004) [131] | Peer reviewed article | Not research | People who experienced a natural disaster | Not specified | Natural disaster | Not applicable | Design studies to investigate decision making capacity, publish on how capacity was determined, participants with insufficient decision making capacity, and predictors of capacity. Study decision-making among those with acute stress disorder and post-traumatic stress disorder to learn about differential sensitivity to re-traumatization. During long-term follow-up, collect data on participant reactions to participation over time. |
| Satapathy et al. (2020) [132] | Peer reviewed article | Review | Children who have experienced trauma | Childhood | Not specified | India, Norway, Australia, Canada, United States | Because resilience is a multi-faceted concept, design resilience scales to address resilience more fully and be specific about what aspects of resilience you are addressing. Conduct longitudinal studies, and investigate divergent validity and sensitivity and specificity. Because resilience is influenced by the environment develop resilience scales for specific societies. Create measures for children and study the phenomenology of resilience. |
| Tol (2020) [133] | Peer reviewed article | Not research | People who have experienced interpersonal violence | Childhood and adulthood | Interpersonal violence | Not specified | If you are researching interpersonal violence and mental health use a social justice frame. This research must be placed within a larger context, and a social justice frame would promote understanding multiple aspects and interrelationships of wellbeing, as well as unite research and practice efforts. |
| Van der Velden, Bosmans, & Scherpenzeel (2013) [134] | Peer reviewed article | Cohort | Danish panel for online survey responses | Childhood and adulthood | Multiple (Traumatic Events Scale) | Denmark | Know that while participants experience questions on trauma as less pleasant than questions that are not on trauma, they also perceive questions on trauma as opportunities for them to be reflective. Demographic factors, having PTSD,^iii^ personality, and coping self-efficacy may be associated with how participants perceive questions on trauma, but potentially not as strongly as we may expect. How participants perceive involvement with prior research may be a bigger predictor of how they perceive participation in trauma research. |
| Vidal et al. (2020) [135] | Peer reviewed article | Review | Multisystem-involved youth | Childhood | Not specified | United States | To design effective interventions trauma-informed standardized assessments of multisystem-involved youth is necessary. |

**Selected Recommendations Pertaining to the Nature and Scope of Trauma Research by Article**

| **Article** | **Publication Type** | **Study Type** | **Population** | **When Trauma Occurred** | **Trauma Type** | **Country** | **Recommendations** |
| --- | --- | --- | --- | --- | --- | --- | --- |
| Walsh et al. (2018) [136] | Peer reviewed article | Qualitative | Members of a parent support group and parents of sexually abused children who had a forensic interview; children aged 13 to 17 whose parents participated in the study | Childhood | Sexual | United States | Understand that people may be more willing to participate in trauma research than we assume. Many people feel that participation in research is very important and may not be distressed by participation. |
| Waterman et al. (2019) [137] | Peer reviewed article | Mixed methods | University students in a relationship | Adulthood | Intimate partner violence | United States | Know that experience sampling methodology may measure intimate partner violence more accurately than other methods and is well tolerated by participants. However, participant retention may be a challenge and researchers should think about how to non-coercively increase retention. |
| White et al. (2013) [138] | Peer reviewed article | Review | Women of color who have experienced intimate partner violence | Adulthood | Emotional, physical, sexual | United States | To build capacity and to conduct better research and research that has more impact, get trained in and use CBPR [5] ^ii^. There is a need for CBPR [5] in quantitative studies on trauma with marginalized groups. Seek to develop valid and reliable instruments that are culturally relevant. In-person recruitment may work well when conducting research with marginalized groups; seek input on recruitment methods from community. Seek community input on the best method of data collection and on appropriate participant reimbursements. Consider developing “modules” of research methods that have shared standardization but that can be selected for research with different communities. Create tangible products that benefit the community while you work towards your longer research objective. |

* Additional information from articles, such as outcomes measured, additional recommendations, and study inclusion criteria, where applicable, available upon request

Summary of Number of Articles with Select Recommendations by Applicable Research Stage and Recommendation Category in 2018 and 2020 Review Conducted in the United States on Research with Trauma-Exposed Populations

|  | **Total** | **Community Benefit** | **Participant Benefit** | **Safety** | **Researcher Well-being** | **Nature and Scope of Trauma Research** |
| --- | --- | --- | --- | --- | --- | --- |
| **Research Stage** | **N** | **N** | **N** | **N** | **N** | **N** |
| **Research Design** | **90** | **35** [1, 2, 4, 10-12, 15-18, 20-25, 28-31, 37, 43, 46, 55, 67, 98, 110, 116, 118, 119, 123, 124, 126, 133, 139] | **5** [27, 33, 36, 88, 121] | **19** [1, 35, 43, 46, 47, 51, 56, 58, 61, 62, 65, 67, 69, 73, 87, 92, 120, 140, 141] | **17** [36, 55, 62, 71-77, 82-84, 86-88] | **38** [3, 6, 28, 64, 67, 88, 91, 93, 96, 97, 99, 101, 102, 104, 105, 107, 109-111, 113-116, 118-128, 132, 133, 138, 141] |
| **Recruitment** | **24** | **11** [1, 2, 4, 12, 19, 35, 37, 43, 53, 73, 138] | **2** [33, 58] | **16** [46, 49, 53, 57, 59, 62-64, 66, 67, 69, 73, 92, 97, 120, 131] | **0** | **9** [96, 101, 115, 120, 127, 130, 131, 136, 138] |
| **Consent** | **27** | **7** [4, 12, 23, 28, 37, 43] | **9** [16, 33, 36, 43, 58, 65, 87, 111, 122] | **11** [16, 41, 43, 45, 52, 53, 59, 63, 67, 69, 73] | **0** | **3** [89, 127, 138] |
| **Data Collection** | **65** | **16** [1, 2, 4, 8, 12, 16, 18, 22, 23, 30, 37, 53, 54, 103, 118, 124] | **20** [16, 32-38, 40, 47, 48, 52, 64, 88, 101, 107, 111, 113, 122, 127] | **39** [1, 16, 34, 36, 37, 40, 42-48, 50, 51, 54-60, 62-69, 73, 87, 88, 92, 97, 113, 114, 120, 122] | **11** [36, 37, 65, 74-77, 81, 82, 84, 85] | **33** [16, 36, 40, 43, 47, 50, 64, 88, 90, 92, 95, 97, 99-101, 103, 106, 107, 111, 113, 114, 117, 118, 120, 122, 125, 127, 130, 131, 134, 135, 137, 138] |
| **Results Dissemination** | **34** | **17** [4, 7, 10, 12, 20, 23-25, 30, 31, 34, 37, 43, 46, 67, 124, 138] | **6** [32-34, 40, 58, 122] | **4** [43, 59, 67, 122] | **1** [74] | **6** [111, 124, 125, 127, 131, 138] |

*Note some articles had recommendations pertaining to multiple recommendation categories and are reported as such in this table; the prior tables present articles within one recommendation category.

Select Gaps for Public Health Practitioners to be Able to Plan and Implement Trauma-Informed Research Studies Identified in 2018 and 2020 Review Conducted in the United States on Research with Trauma-Exposed Populations

**Research Design and Conduct**

| **Gap** | **Explanation and select questions to consider** |
| --- | --- |
| Theory | There is little advice on what role theory should play in trauma-informed research design. Guidelines ought to address differences between theory and both theoretical and conceptual frameworks in relation to the conduct of trauma-informed research, the applicability of specific theory to trauma-informed research, effective operationalization of theory in trauma-informed research, and gaps in existing theoretical knowledge [142, 143]. Future authors should consider the development and adaptation of theories to guide **all** stages of trauma-informed public health research. Moreover, theories used by researchers should allow for a broad understanding of trauma, including historical trauma.   - How can we break down disciplinary silos with regard to which theories and frameworks trauma researchers use? Public health researchers should have access to theoretical contributions on trauma from across a range of disciplines, yet frequently which theories or frameworks trauma researchers use is fragmented (e.g., those using the structural theory of dissociation [144] often do not also use a historical trauma [145, 146] framework) - How can we use theory to better understand the relationship between types of trauma exposures and specific health outcomes? - How can we use theory to identify intervention points for secondary prevention (e.g., after trauma has occurred but before health impacts are manifested)? |
| Defining and investigating research populations | We also note that there is a lack of guidance on which populations are meaningful to identify in trauma research, and how these populations ought to be investigated. We see the lack of guidance on this topic as a deficit for both research on trauma exposure and trauma interventions. Identifying populations in relation to trauma exposures or effects may be particularly helpful in intervention research: for example, evidence supports the effectiveness of exposure therapy for PTSD [147], yet many clinicians assert that exposure therapy can be harmful for people with complex PTSD [148]. When identifying sub-populations, trauma researchers may consider culture, trauma exposure or effects, and exposure to racism and other systematic oppressions (also forms of trauma, we would argue; see ‘systemic issues’ below too). Developing guidelines on addressing culture in trauma research [149, 150], including the adoption of cultural humility [13] practices, can help researchers conduct studies relevant to trauma-exposed populations and account for how culture may shape the manifestation of trauma symptoms.   - How do we define what communities are trauma-exposed? - Should we develop a consensus on communities to be prioritized in research? Are there other ways to encourage research on the causes, prevention, and treatment of trauma that affects populations or communities? - What supports are necessary to ensure the representation of people of color and other marginalized groups in research? - How do we define and investigate trauma exposure comprehensively (e.g., beyond interpersonal interactions that carry severe threat of harm)?   Clinical interest questions:   - Research on trauma exposure or effects does not often identify participants with trauma-related dissociative disorders, even though some studies suggest that these conditions may be common [151-156].   - How do we encourage public health researchers to account for trauma-related dissociative disorders when conducting formative trauma research or studying health services for trauma-exposed populations?   - How do we specifically encourage more epidemiological studies on dissociative disorders (e.g., prevalence, mental and physical health comorbidities and effects, as well as genetic and environmental etiology [157])?   - What are the potential costs of not accounting for and not studying the epidemiology of trauma-related dissociative disorders? |

Select Gaps for Public Health Practitioners to be Able to Plan and Implement Trauma-Informed Research Studies Identified in 2018 and 2020 Review Conducted in the United States on Research with Trauma-Exposed Populations

**Systemic Issues**

| **Gap** | **Explanation and select questions to consider** |
| --- | --- |
| Multi-level frameworks | Utilizing multi-level frameworks in public health research on trauma could promote scholarship on antecedents and sources of trauma (e.g., neighborhood exposures, historical effects, etc.), as well as the design and evaluation of effective trauma interventions.   - What practices can help trauma researchers investigate trauma beyond interpersonal interactions and intrapersonal characteristics (e.g., investigate collective and community trauma [158])? |
| Multiple dimensions of trauma | There are rich bodies of literature on both the psychological effects of trauma and on historical trauma, but this literature is not unified. Developing a more unified and nuanced multi-dimensional understanding of trauma could promote theoretical advances and encourage researchers to more comprehensively addresses trauma exposures and effects. This could improve the design and evaluation of trauma interventions. A multi-level and more multi-dimensional understanding of trauma may also help professionals better identify sub-populations with unique trauma exposures.   - How should we measure and operationalize historical and other forms of collective trauma [159], and what measures would be best in what contexts? - In what ways could we assess and account for what we might call “collective dissociation” (e.g., double consciousness [160] and cultural fragmentation)? - How can we encourage researchers to appropriately assess and account for multiple forms of individual-level dissociation in trauma research, including identity fragmentation, in order to improve the validity of their results? - How should researchers best account for chronic “low-level” stressors, such as “micro”-aggressions, that may cumulatively constitute trauma and that may interact with more-recognized traumas to affect health? |
| Systemic injustices | There is both a need for increased studies on as well as guidance on how to conduct studies with those who have experienced systemic injustices or abuses of institutional power. For example, literature suggests that racism can create race-based stress or racial trauma [161-167]. Due to its systematic nature, racism and other systemic oppressions are experienced cumulatively across the life course [168] and during critical developmental periods [168, 169]. Also, exposure to racism may affect both the direct result that trauma may have on someone, as well as the availability of resources to handle trauma [170, 171]. Both merit additional attention from public health researchers. People who have experienced abuses of institutional power include refugee children who have been separated from their caregivers and those who have lost someone due to police violence. It may be particularly important to focus on children who have been separated as they comprise a cohort in need of services and support. We believe that interventions and services for people who have experienced abuses of institutional power may be more effective if they acknowledge or address the structural issues that allowed the abuse of power to occur.   - How should researchers address or incorporate an understanding of racism and colonialism as forms of trauma in their research? - How can we foster research on interventions geared towards refugee children who have been separated from their parents, and may have been otherwise abused or sex trafficked, that will be responsive to their needs now and when they begin to seek out services as young adults? - How can we design intervention research that is geared specifically towards the needs of children who have lost a family member or friend to police violence? The needs of these children may be great, and they are a relatively small number and geographically spread out. In designing interventions for these children, what are key components to be addressed (e.g., besides dealing the effects of the actual trauma there may also be a need to address societal responses of victim blaming and not holding officers accountable; there may also need to be a component that focuses on addressing parent needs and bereavement.) |

Select Gaps for Public Health Practitioners to be Able to Plan and Implement Trauma-Informed Research Studies Identified in 2018 and 2020 Review Conducted in the United States on Research with Trauma-Exposed Populations

**Research Translation/Applicability**

| **Gap** | **Explanation and select questions to consider** |
| --- | --- |
| Scale-up | The scale-up of interventions does not pertain strictly to trauma prevention and care, but there is a particularly immense need for literature to address scaling up evidenced-based and promising interventions, practices, and standards of care that are trauma-specific [172]. To support scaling up evidence-based or promising interventions, scholars can consider intervention creation and adaptation practices that support replication, such as identifying essential and modifiable intervention/program components [173-176].   - What intervention creation and adaptation practices are going to be most effective for scaling up evidence-based and promising trauma interventions, and for enhancing translatability of findings to wider audiences, including mental health practitioners? - How can we encourage more researchers to examine which program/intervention components are essential to outcomes across environments, and which may have to be modified in different environments or to different populations? - What are best practices trauma intervention researchers can use to educate the public on their findings, in order to promote public investment in trauma prevention and treatment research? |
| Policy | Consideration of policy also is not specific to trauma research but at present it is pertinent to trauma research (e.g., discussion on routine screening for adverse childhood effects [177-178]; see Finkelhor, 2018 [179] and Dowd, 2019 [180]). In considering how to design or use research to impact policy, researchers may benefit from increased training on policy research principles, as well as from training on building relationships with communities who are or may be affected by the presence or lack of a policy, policy makers, and other stakeholders, such as independent trauma-focused organizations. Initial templates for addressing intersections of public health research and policy may be obtained from the Robert Wood Johnson Foundation (e.g., Policies for Action) [181].   - What processes can researchers use to identify meaningful policy intervention points to prevent and reduce the impact of trauma? - How can researchers appropriately engage in policy and work to promote equitable policy formation, such that those affected by trauma are included in making policy? - What research design methods are most effective for impacting policy and under what conditions? |


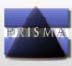
**PRISMA 2009 Checklist**

| **Section/topic** | **#** | **Checklist item** | **Reported on page #** |
| --- | --- | --- | --- |
| **TITLE** | | |  |
| Title | 1 | Identify the report as a systematic review, meta-analysis, or both. | 1 |
| **ABSTRACT** | | |  |
| Structured summary | 2 | Provide a structured summary including, as applicable: background; objectives; data sources; study eligibility criteria, participants, and interventions; study appraisal and synthesis methods; results; limitations; conclusions and implications of key findings; systematic review registration number. | 2 |
| **INTRODUCTION** | | |  |
| Rationale | 3 | Describe the rationale for the review in the context of what is already known. | 3-5 |
| Objectives | 4 | Provide an explicit statement of questions being addressed with reference to participants, interventions, comparisons, outcomes, and study design (PICOS). | 5 |
| **METHODS** | | |  |
| Protocol and registration | 5 | Indicate if a review protocol exists, if and where it can be accessed (e.g., Web address), and, if available, provide registration information including registration number. | 5-6 |
| Eligibility criteria | 6 | Specify study characteristics (e.g., PICOS, length of follow-up) and report characteristics (e.g., years considered, language, publication status) used as criteria for eligibility, giving rationale. | 5-6 |
| Information sources | 7 | Describe all information sources (e.g., databases with dates of coverage, contact with study authors to identify additional studies) in the search and date last searched. | 5 |
| Search | 8 | Present full electronic search strategy for at least one database, including any limits used, such that it could be repeated. | S1 |
| Study selection | 9 | State the process for selecting studies (i.e., screening, eligibility, included in systematic review, and, if applicable, included in the meta-analysis). | 6-7 |
| Data collection process | 10 | Describe method of data extraction from reports (e.g., piloted forms, independently, in duplicate) and any processes for obtaining and confirming data from investigators. | 7-9 |
| Data items | 11 | List and define all variables for which data were sought (e.g., PICOS, funding sources) and any assumptions and simplifications made. | 5-9 |
| Risk of bias in individual studies | 12 | Describe methods used for assessing risk of bias of individual studies (including specification of whether this was done at the study or outcome level), and how this information is to be used in any data synthesis. | NA |
| Summary measures | 13 | State the principal summary measures (e.g., risk ratio, difference in means). | NA |
| Synthesis of results | 14 | Describe the methods of handling data and combining results of studies, if done, including measures of consistency (e.g., I^2^) for each meta-analysis. | 5-9; I^2^ NA |

Page 1 of 2

| **Section/topic** | **#** | **Checklist item** | **Reported on page #** |
| --- | --- | --- | --- |
| Risk of bias across studies | 15 | Specify any assessment of risk of bias that may affect the cumulative evidence (e.g., publication bias, selective reporting within studies). | NA |
| Additional analyses | 16 | Describe methods of additional analyses (e.g., sensitivity or subgroup analyses, meta-regression), if done, indicating which were pre-specified. | 5-9 |
| **RESULTS** | | |  |
| Study selection | 17 | Give numbers of studies screened, assessed for eligibility, and included in the review, with reasons for exclusions at each stage, ideally with a flow diagram. | 9-10 |
| Study characteristics | 18 | For each study, present characteristics for which data were extracted (e.g., study size, PICOS, follow-up period) and provide the citations. | S2 |
| Risk of bias within studies | 19 | Present data on risk of bias of each study and, if available, any outcome level assessment (see item 12). | NA |
| Results of individual studies | 20 | For all outcomes considered (benefits or harms), present, for each study: (a) simple summary data for each intervention group (b) effect estimates and confidence intervals, ideally with a forest plot. | NA |
| Synthesis of results | 21 | Present results of each meta-analysis done, including confidence intervals and measures of consistency. | 11-18 Synthesis; meta-analysis NA |
| Risk of bias across studies | 22 | Present results of any assessment of risk of bias across studies (see Item 15). | NA |
| Additional analysis | 23 | Give results of additional analyses, if done (e.g., sensitivity or subgroup analyses, meta-regression [see Item 16]). | NA |
| **DISCUSSION** | | |  |
| Summary of evidence | 24 | Summarize the main findings including the strength of evidence for each main outcome; consider their relevance to key groups (e.g., healthcare providers, users, and policy makers). | 21-24; strength of evidence NA |
| Limitations | 25 | Discuss limitations at study and outcome level (e.g., risk of bias), and at review-level (e.g., incomplete retrieval of identified research, reporting bias). | 22-23 |
| Conclusions | 26 | Provide a general interpretation of the results in the context of other evidence, and implications for future research. | 23-s24 |
| **FUNDING** | | |  |
| Funding | 27 | Describe sources of funding for the systematic review and other support (e.g., supply of data); role of funders for the systematic review. | 25 |

*From:*  Moher D, Liberati A, Tetzlaff J, Altman DG, The PRISMA Group (2009). Preferred Reporting Items for Systematic Reviews and Meta-Analyses: The PRISMA Statement. PLoS Med 6(7): e1000097. doi:10.1371/journal.pmed1000097

For more information, visit: **www.prisma-statement.org**.

Page 2 of 2

**REFERENCES**

1. Ahrens CE, Isas L, Viveros M. Enhancing Latinas’ participation in research on sexual assault: Cultural considerations in the design and implementation of research in the Latino community. Violence Against Women. 2011;17(2):177-88.

2. Allodi FA. Assessment and treatment of torture victims: a critical review. Journal of Nervous and Mental Disease. 1991.

3. Alvarez A. Seeing race in the research on youth trauma and education: A critical review. Review of Educational Research. 2020.

4. Anderson ML, Craig W, Kelly S, Ziedonis DM. Deaf people’s help-seeking following trauma: Experiences with and recommendations for the Massachusetts behavioral health care system. Psychological Trauma: Theory, Research, Practice, and Policy. 2017;9(2):239.

5. Israel BA, Schulz AJ, Parker EA, Becker AB, Allen AJ, Guzman JR, et al. Critical issues in developing and following CBPR principles. Community-based participatory research for health: Advancing social and health equity. 2017;3:32-5.

6. Andrews NCZ, Pepler DJ, Motz M. Research and Evaluation With Community-Based Projects: Approaches, Considerations, and Strategies. American Journal of Evaluation. 2019;40(4):548-61.

7. Avey JP, Hiratsuka VY, Dirks LG, Moore LA, Beach B, Novins DK, et al. DISSEMINATING INFORMATION ON TRAUMA SCREENING, BRIEF INTERVENTION, AND REFERRAL TO TREATMENT IN A TRIBAL HEALTH SETTING: A CASE STUDY. American Indian and Alaska native mental health research (Online). 2018;25(1):43.

8. Barrios VR, Khaw LBL, Bermea A. Future Directions in Intimate Partner Violence Research: An Intersectionality Framework for Analyzing Women's Processes of Leaving Abusive Relationships. Journal of …. 2020.

9. Crenshaw K. Demarginalizing the intersection of race and sex: A black feminist critique of antidiscrimination doctrine, feminist theory and antiracist politics. u Chi Legal f. 1989:139.

10. Beharry M, Harpin SB, AlMakadma A, Ammerman S, Eisenstein E, Warf C, et al. The healthcare needs and rights of youth experiencing homelessness society for adolescent health and medicine. JOURNAL OF ADOLESCENT HEALTH. 2018;63(3):372-5.

11. Benevides TW, Shore SM, Palmer K, Duncan P. Listening to the autistic voice: Mental health priorities to guide research and practice in autism from a stakeholder-driven project. …. 2020.

12. Caldwell JY, Davis JD, Du Bois B, Echo-Hawk H, Erickson JS, Goins RT, et al. Culturally competent research with American Indians and Alaska Natives: findings and recommendations of the first symposium of the work group on American Indian Research and Program Evaluation Methodology. American Indian and Alaska Native Mental Health Research: The Journal of the National Center. 2005;12(1):1-21.

13. Tervalon M, Murray-Garcia J. Cultural humility versus cultural competence: A critical distinction in defining physician training outcomes in multicultural education. Journal of health care for the poor and underserved. 1998;9(2):117-25.

14. Edwards Y. Healing the soul wound: The retraditionalization of Native Americans in substance abuse treatment. 2003.

15. Chamberlain C, Gee G, Brown SJ, Atkinson J, Herrman H, Gartland D, et al. Healing the Past by Nurturing the Future - Co-designing perinatal strategies for Aboriginal and Torres Strait Islander parents experiencing complex trauma: Framework and protocol for a community-based participatory action research study. BMJ Open. 2019;9(6).

16. Downes J, Kelly L, Westmarland N. Ethics in violence and abuse research-a positive empowerment approach. Sociological Research Online. 2014;19(1):1-13.

17. Ghanbarpour S, Palotai A, Kim ME, Aguilar A, Flores J, Hodson A, et al. An exploratory framework for community-led research to address intimate partner violence: A case study of the survivor-centered advocacy project. Journal of family violence. 2018;33(8):521-35.

18. Hamby S, Schultz K, Elm J. Understanding the burden of trauma and victimization among American Indian and Alaska native elders: historical trauma as an element of poly-victimization. J Trauma Dissociation. 2020;21(2):172-86.

19. Hebenstreit CL, DePrince AP. Perceptions of participating in longitudinal trauma research among women exposed to intimate partner abuse. Journal of Empirical Research on Human Research Ethics. 2012;7(2):60-9.

20. Holmes EA, Connor RCO, Perry VH, Tracey I. Multidisciplinary research priorities for the COVID-19 pandemic: a call for action for mental health science. The Lancet …. 2020.

21. Javakhishvili JD, Ardino V, Bragesjö M, Kazlauskas E, Olff M, Schäfer I. Trauma-informed responses in addressing public mental health consequences of the COVID-19 pandemic: position paper of the European Society for Traumatic Stress Studies (ESTSS). European Journal of Psychotraumatology. 2020;11(1).

22. Leung E, Flanagan T. Let's do this together: an integration of photovoice and mobile interviewing in empowering and listening to LGBTQ+ youths in context. International Journal of Adolescence and Youth. 2019;24(4):497-510.

23. Leung L, Miedema S, Warner X, Homan S. Making feminism count: integrating feminist research principles in large-scale quantitative research on violence against women and girls. Gender & …. 2019.

24. McCauley HL, Campbell R. Advancing theory, methods, and dissemination in sexual violence research to build a more equitable future: an intersectional, community-engaged approach. Violence against …. 2019.

25. Nnawulezi N, Sullivan CM, Marcus S. Negotiating participatory research processes with domestic violence program staff to obtain ecologically valid data. Journal of …. 2019.

26. Quina K, Rose JS, Harlow LL, Morokoff PJ, Deiter PJ, Whitmire LE, et al. Focusing on participants: Feminist process model for survey modification. Psychology of Women Quarterly. 1999;23(3):459-83.

27. Roche P, Shimmin C, Hickes S, Khan M, Sherzoi O, Wicklund E, et al. Valuing All Voices: refining a trauma-informed, intersectional and critical reflexive framework for patient engagement in health research using a qualitative descriptive approach. Res Involv Engagem. 2020;6:42.

28. Thomas SN, Weber S, Bradbury-Jones C. Using Participatory and Creative Methods to Research Gender-Based Violence in the Global South and With Indigenous Communities: Findings From a Scoping Review. Trauma, violence & abuse. 2020:1524838020925775.

29. Turpel-Lafond ME, Chondoma L. BUILDING INDIGENOUS-LED ENGAGEMENT FRAMEWORKS. 2019.

30. Twis MK, Preble K. Intersectional standpoint methodology: Toward theory-driven participatory research on human trafficking. Violence and Victims. 2020.

31. Wright NM, Olomi JM, DePrince AP. Community-Engaged Research: Exploring a Tool for Action and Advocacy. Journal of Trauma & Dissociation. 2020;21(4):452-67.

32. Burgess-Proctor A, editor Methodological and ethical issues in feminist research with abused women: Reflections on participants' vulnerability and empowerment. Women's Studies International Forum; 2015: Elsevier.

33. Campbell R, Goodman-Williams R, Javorka M. A Trauma-Informed Approach to Sexual Violence Research Ethics and Open Science. J Interpers Violence. 2019;34(23-24):4765-93.

34. Gekoski A, Gray JM, Adler JR. Interviewing women bereaved by homicide: assessing the impact of trauma-focused research. Psychology, crime & law. 2012;18(2):177-89.

35. Logan T, Walker R, Shannon L, Cole J. Combining ethical considerations with recruitment and follow-up strategies for partner violence victimization research. Violence Against Women. 2008;14(11):1226-51.

36. Paton J, Horsfall D, Carrington A. Sensitive Inquiry in Mental Health: A Tripartite Approach. International Journal of Qualitative Methods. 2018;17(1):1609406918761422.

37. Pk S. Our seat at the table: Mentorship, advocacy, & youth leadership in qualitative research. Journal of family violence. 2018;33(8):579-85.

38. Raghavan S, Sandanapitchai P. The relationship between cultural variables and resilience to psychological trauma: A systematic review of the literature. Traumatology. 2020.

39. Edition F. Diagnostic and statistical manual of mental disorders. American Psychiatric Association2013.

40. Wager NM. Respondents’ experiences of completing a retrospective web-based, sexual trauma survey: Does a history of sexual victimization equate with risk for harm? Violence and victims. 2012;27(6):991-1004.

41. Ahlin EM. Moving beyond prison rape: Assessing sexual victimization among youth in custody. Aggression and Violent Behavior. 2019.

42. Allard CB, Straus E, Ra MI, Thomas KB. Japanese students do see the value of asking about child abuse and trauma in the research setting. … empirical research …. 2019.

43. Allden K, Jones L, Weissbecker I, Wessells M, Bolton P, Betancourt TS, et al. Mental health and psychosocial support in crisis and conflict: report of the Mental Health Working Group. Prehospital and Disaster Medicine. 2009;24(S2):s217-s27.

44. Black MC, Kresnow M-j, Simon TR, Arias I, Shelley G. Telephone survey respondents’ reactions to questions regarding interpersonal violence. Violence and Victims. 2006;21(4):445-59.

45. Boscarino JA, Figley CR, Adams RE, Galea S, Resnick H, Fleischman AR, et al. Adverse reactions associated with studying persons recently exposed to mass urban disaster. The Journal of nervous and mental disease. 2004;192(8):515.

46. Bowen EA, Murshid NS. Trauma-informed social policy: A conceptual framework for policy analysis and advocacy. American journal of public health. 2016;106(2):223-9.

47. Brown VM, Strauss JL, LaBar KS, Gold AL, McCarthy G, Morey RA. Acute effects of trauma-focused research procedures on participant safety and distress. Psychiatry research. 2014;215(1):154-8.

48. Campbell R, Adams AE, Wasco SM, Ahrens CE, Sefl T. Training interviewers for research on sexual violence: A qualitative study of rape survivors' recommendations for interview practice. Violence against women. 2009;15(5):595-617.

49. Campbell R, Adams AE. Why do rape survivors volunteer for face-to-face interviews? A meta-study of victims' reasons for and concerns about research participation. Journal of Interpersonal violence. 2009;24(3):395-405.

50. Campbell R, Adams AE, Wasco SM, Ahrens CE, Sefl T. “What has it been like for you to talk with me today?”: The impact of participating in interview research on rape survivors. Violence against women. 2010;16(1):60-83.

51. Chang JC, Decker MR, Moracco KE, Martin SL, Petersen R, Frasier PY. Asking about intimate partner violence: advice from female survivors to health care providers. Patient education and counseling. 2005;59(2):141-7.

52. Copes H, Tchoula W, Brookman F, Ragland J. Photo-elicitation interviews with vulnerable populations: Practical and ethical considerations. Deviant Behavior. 2018;39(4):475-94.

53. Dehghan R, Wilson J. Healthcare professionals as gatekeepers in research involving refugee survivors of sexual torture: An examination of the ethical issues. Developing world bioethics. 2019;19(4):215-23.

54. Douglas EM, Hines DA, Dixon L, Celi EM, Lysova AV. Using technology to conduct focus groups with a hard-to-reach population: a methodological approach concerning male victims of partner abuse in four English-speaking countries. Journal of interpersonal violence. 2018:0886260518799459.

55. Goodwin J, Tiderington E. Building trauma-informed research competencies in social work education. Social Work Education.14.

56. Guerra C, Pereda N. Research with adolescent victims of child sexual abuse: Evaluation of emotional impact on participants. Journal of child sexual abuse. 2015;24(8):943-58.

57. Hardesty JL, Haselschwerdt ML, Crossman KA. Qualitative Research on Interpersonal Violence: Guidance for Early Career Scholars. Journal of Interpersonal Violence. 2019;34(23-24):4794-816.

58. Jorm AF, Kelly CM, Morgan AJ. Participant distress in psychiatric research: A systematic review. Psychological medicine. 2007;37(7):917-26.

59. Kyegombe N, Banks LM, Kelly S, Kuper H, Devries KM. How to conduct good quality research on violence against children with disabilities: key ethical, measurement, and research principles. BMC Public Health. 2019;19(1).

60. Kimberg LS. Addressing intimate partner violence with male patients: a review and introduction of pilot guidelines. Journal of general internal medicine. 2008;23(12):2071-8.

61. Linabary JR, Corple DJ. Privacy for whom?: A feminist intervention in online research practice. Information, Communication & Society. 2019.

62. Mwambari D. Local positionality in the production of knowledge in Northern Uganda. International Journal of Qualitative Methods. 2019.

63. Pickles J. Including and involving young people (under 18's) in hate research without the consent of parents. Qualitative Research. 2020;20(1):22-38.

64. Rivlin A, Marzano L, Hawton K, Fazel S. Impact on prisoners of participating in research interviews related to near-lethal suicide attempts. Journal of affective disorders. 2012;136(1-2):54-62.

65. Scerri CS, Abela A, Vetere A. Ethical dilemmas of a clinician/researcher interviewing women who have grown up in a family where there was domestic violence. International journal of qualitative methods. 2012;11(2):102-31.

66. Schwerdtfeger KL. The appraisal of quantitative and qualitative trauma-focused research procedures among pregnant participants. Journal of empirical research on human research ethics. 2009;4(4):39-51.

67. Sullivan CM, Cain D. Ethical and safety considerations when obtaining information from or about battered women for research purposes. Journal of interpersonal violence. 2004;19(5):603-18.

68. Testa M, Livingston JA, VanZile-Tamsen C. Advancing the study of violence against women using mixed methods: Integrating qualitative methods into a quantitative research program. Violence against women. 2011;17(2):236-50.

69. Waechter R, Kumanayaka D, Angus-Yamada C, Wekerle C, Smith S. Maltreatment history, trauma symptoms and research reactivity among adolescents in child protection services. Child and adolescent psychiatry and mental health. 2019;13(1):1-10.

70. Adams JR. A Qualitative Descriptive Study on Compassion Fatigue with Male Mental Health Professionals Who Treat Trauma Survivors: search.proquest.com; 2020.

71. Berger R. Studying trauma: Indirect effects on researchers and self-And strategies for addressing them. European Journal of Trauma & Dissociation. 2020.

72. Berger R, Quiros L. Best practices for training trauma-informed practitioners: Supervisors’ voice. Traumatology. 2016;22(2):145.

73. Btoush R, Campbell JC. Ethical conduct in intimate partner violence research: Challenges and strategies. Nursing outlook. 2009;57(4):210-6.

74. Coles J, Mudaly N. Staying safe: Strategies for qualitative child abuse researchers. Child Abuse Review: Journal of the British Association for the Study and Prevention of Child Abuse and Neglect. 2010;19(1):56-69.

75. Coles J, Astbury J, Dartnall E, Limjerwala S. A qualitative exploration of researcher trauma and researchers’ responses to investigating sexual violence. Violence against women. 2014;20(1):95-117.

76. Eadesa AM, Hacketta M, Ravenc M, Liua H. The impact of vicarious trauma on Aboriginal and/or Torres Strait Islander health researchers. Public Health Research …. 2020.

77. Fohring S. The risks and rewards of researching victims of crime. Methodological Innovations. 2020.

78. Institute H. Headington Institute 2021 [Available from: <https://www.headington-institute.org/>.

79. Science AW. Sexual Violence Research Initiative 2021 [Available from: [www.svri.org](file:///C:\Users\kevin\Dropbox\My%20PC%20(LAPTOP-79F0B9ES)\Downloads\www.svri.org).

80. Guerzoni MA. Vicarious trauma and emotional labour in researching child sexual abuse and child protection: A postdoctoral reflection. Methodological Innovations. 2020.

81. Jeftic A. 'I Was Close to Them': Re-experiencing War through Trauma-based Interviews. Social Epistemology. 2020;34(1):79-85.

82. Markowitz A. The better to break and bleed with: Research, violence, and trauma. Geopolitics. 2021.

83. Nikischer A. Vicarious trauma inside the academe: Understanding the impact of teaching, researching and writing violence: Springer; 2019.

84. Smith AM, Hamilton AB, Loeb T, Pemberton J, Wyatt GE. Reactions of Novice Interviewers Conducting Trauma Research With Marginalized Communities: A Qualitative Analysis. Journal of Interpersonal Violence.

85. Starcher D, Stolzenberg SN. Burnout and secondary trauma among forensic interviewers. Child & Family Social Work. 2020;25(4):924-34.

86. Taylor J, Bradbury‐Jones C, Breckenridge JP, Jones C, Herber OR. Risk of vicarious trauma in nursing research: a focused mapping review and synthesis. Journal of clinical nursing. 2016;25(19-20):2768-77.

87. Cordisco Tsai L. Conducting research with survivors of sex trafficking: Lessons from a financial diaries study in the Philippines. British Journal of Social Work. 2018;48(1):158-75.

88. Wager N. Researching sexual revictimisation: associated ethical and methodological issues, and possible solutions. Child Abuse Review. 2011;20(3):158-72.

89. Abu-Rus A, Bussell N, Olsen DC. Informed consent content in research with survivors of psychological trauma. Ethics & …. 2019.

90. Acierno R, Resnick H, Kilpatrick D, Stark-Riemer W. Assessing elder victimization. Social psychiatry and psychiatric epidemiology. 2003;38(11):644-53.

91. Alaggia R, Collin-Vézina D. Facilitators and barriers to child sexual abuse (CSA) disclosures: A research update (2000–2016). Trauma, Violence, & …. 2019.

92. Baumgartner JN, Kaaya S, Karungula H, Kaale A, Headley J, Tolley E. Domestic violence among adolescents in HIV prevention research in Tanzania: participant experiences and measurement issues. Maternal and child health journal. 2015;19(1):33-9.

93. Berg MT, Mulford CF. Reappraising and redirecting research on the victim–offender overlap. Trauma, Violence, & Abuse. 2020.

94. Binion K, Gray MJ. Minority Stress Theory and Internalized Homophobia among LGB Sexual Assault Survivors: Implications for Posttraumatic Adjustment. Journal of Loss & Trauma.

95. Blasbalg U, Hershkowitz I, Lamb ME, Karni-Visel Y, Ahern EC. Is interviewer support associated with the reduced reluctance and enhanced informativeness of alleged child abuse victims? Law Hum Behav. 2019;43(2):156-65.

96. Boals A, Contractor AA, Blumenthal H. The Utility of College Student Samples in Research on Trauma and Posttraumatic Stress Disorder: A Critical Review. Journal of Anxiety Disorders. 2020.

97. Bouchard KL. Anonymity as a double-edge sword: Reflecting on the implications of online qualitative research in studying sensitive topics. The Qualitative Report. 2016;21(1):59-67.

98. Carr S. 'I am not your nutter': a personal reflection on commodification and comradeship in service user and survivor research. Disability & Society. 2019.

99. Bennett Cattaneo L, Chapman AR. Risk assessment with victims of intimate partner violence: Investigating the gap between research and practice. Violence against women. 2011;17(10):1286-98.

100. Cederborg AC, Lamb M. Interviewing alleged victims with intellectual disabilities. Journal of Intellectual Disability Research. 2008;52(1):49-58.

101. Chae Y, Goodman GS, Bederian-Gardner D, Lindsay A. Methodological issues and practical strategies in research on child maltreatment victims’ abilities and experiences as witnesses. Child Abuse and Neglect-the International Journal. 2011;35(4):240.

102. Chan TS, Teram E, Shaw I. Balancing methodological rigor and the needs of research participants: A debate on alternative approaches to sensitive research. Qualitative Health Research. 2017;27(2):260-70.

103. Chozinski BA, Gonzalez A. Using georeferenced photo-elicitation projects to understand survivor resources: a method for trauma-informed practice in higher education. Journal of American College Health. 2020.

104. Clark CJ, Shahrouri M, Halasa L, Khalaf I, Spencer R, Everson-Rose S. A mixed methods study of participant reaction to domestic violence research in Jordan. Journal of interpersonal violence. 2012;27(9):1655-76.

105. Clark JJ, Walker R. Research ethics in victimization studies: Widening the lens. Violence against women. 2011;17(12):1489-508.

106. Collings SJ. A proposed model for evaluating the impact of participating in trauma-focused research. South African journal of psychology. 2019.

107. Decker SE, Naugle AE, Carter-Visscher R, Bell K, Seifert A. Ethical issues in research on sensitive topics: Participants' experiences of distress and benefit. Journal of empirical research on human research ethics. 2011;6(3):55-64.

108. Newman E, Willard T, Sinclair R, Kaloupek D. Empirically supported ethical research practice: The costs and benefits of research from the participants’ view. Accountability in Research. 2001;8(4):309-29.

109. Danese A. Annual Research Review: Rethinking childhood trauma‐new research directions for measurement, study design and analytical strategies. Journal of Child Psychology and Psychiatry. 2020.

110. Dell NA, Maynard BR, Born KR. Helping survivors of human trafficking: A systematic review of exit and postexit interventions. Trauma, Violence …. 2019.

111. DePrince AP, Chu A. Perceived benefits in trauma research: Examining methodological and individual difference factors in responses to research participation. Journal of Empirical Research on Human Research Ethics. 2008;3(1):35-47.

112. DePrince AP, Freyd JJ. Costs and benefits of being asked about trauma history. Journal of Trauma Practice. 2006;3(4):23-35.

113. Draucker CB, Martsolf DS, Poole C. Developing distress protocols for research on sensitive topics. Archives of psychiatric nursing. 2009;23(5):343-50.

114. Finkelhor D, Vanderminden J, Turner H, Hamby S, Shattuck A. Upset among youth in response to questions about exposure to violence, sexual assault and family maltreatment. Child abuse & neglect. 2014;38(2):217-23.

115. Gagnon KL, DePrince AP, Srinivas T, Hasche LK. Perceptions of participation in trauma research among older adults. Traumatology. 2015;21(3):237.

116. Gaillard JC, Peek L. Disaster-zone research needs a code of conduct: nature.com; 2019.

117. Galea S, Nandi A, Stuber J, Gold J, Acierno R, Best CL, et al. Participant reactions to survey research in the general population after terrorist attacks. Journal of Traumatic Stress: Official Publication of The International Society for Traumatic Stress Studies. 2005;18(5):461-5.

118. Golden T. Innovation and Equity in Public Health Research: Testing Arts-Based Methods for Trauma-Informed, Culturally-Responsive Inquiry: core.ac.uk; 2019.

119. Gonçalves M, Matos M. Interpersonal violence in immigrant women in Portugal: An Intersectional approach. Journal of Immigrant & Refugee Studies. 2020.

120. Goodkind JR, Amer S, Christian C, Hess JM, Bybee D, Isakson BL, et al. Challenges and innovations in a community-based participatory randomized controlled trial. Health Education & Behavior. 2017;44(1):123-30.

121. Gultekin L, Kusunoki Y, Sinko L, Cannon L. The Eco‐Social Trauma Intervention Model. Public Health …. 2019.

122. Hoover SM, Morrow SL. Qualitative Researcher Reflexivity: A Follow-Up Study with Female Sexual Assault Survivors. Qualitative Report. 2015;20(9).

123. Keyes TS, Vogel-Ferguson MB. An Approach for Engaging with a Mixed-Race, Rural Community Using Social Work Values and a Community-Based Participatory Research Framework. Journal of Evidence-Based …. 2019.

124. Kim C, Schmuhl M. Assessment of Research on Intimate Partner Violence (IPV) Among Sexual Minorities in the United States. Trauma Violence & Abuse.

125. Massey C, Widom CS. Reactions to research participation in victims of childhood sexual abuse: a comparison of court-substantiated and retrospectively self-reported cases. Journal of Empirical Research on Human Research Ethics. 2013;8(4):77-92.

126. Matheson C, Weightman E. Research and recovery: Can patient participation in research promote recovery for people with complex post‐traumatic stress disorder, CPTSD? Health Expectations. 2019.

127. Newman E, Kaloupek DG. The risks and benefits of participating in trauma‐focused research studies. Journal of Traumatic Stress: Official Publication of The International Society for Traumatic Stress Studies. 2004;17(5):383-94.

128. O'Brien N. Understanding Alternative Bullying Perspectives Through Research Engagement With Young People. Frontiers in Psychology. 2019;10.

129. Øverlien C, Hellevik PM, Korkmaz S. Young Women’s Experiences of Intimate Partner Violence–Narratives of Control, Terror, and Resistance. Journal of family violence. 2020 Nov;35(8):803-14.

130. Pickering CEZ, Maxwell C. Recruitment, enrollment & data collection with victims of elder abuse and neglect identified from police incident reports. J Elder Abuse Negl. 2018;30(5):333-53.

131. Rosenstein DL. Decision‐making capacity and disaster research. Journal of Traumatic Stress: Official Publication of The International Society for Traumatic Stress Studies. 2004;17(5):373-81.

132. Satapathy S, Dang S, Sagar R, Dwivedi SN. Resilience in Children and Adolescents Survived Psychologically Traumatic Life Events: A Critical Review of Application of Resilience Assessment Tools for Clinical Referral and Intervention. Trauma Violence & Abuse.13.

133. Tol WA. Interpersonal violence and mental health: a social justice framework to advance research and practice. Global Mental Health. 2020.

134. van der Velden PG, Bosmans MW, Scherpenzeel AC. The burden of research on trauma for respondents: A prospective and comparative study on respondents evaluations and predictors. PloS one. 2013;8(10).

135. Vidal S, Connell CM, Prince DM, Tebes JK. Multisystem-involved youth: A developmental framework and implications for research, policy, and practice. Adolescent Research Review. 2019.

136. Walsh WA, Wolak J, Lounsbury K, Howley S, Lippert T, Thompson Jr L. Lessons learned: conducting research with victims portrayed in sexual abuse images and their parents. Journal of interpersonal violence. 2018;33(24):3829-39.

137. Waterman EA, Edwards KM, Dardis CM, Kelley EL, Sessarego S. Assessing Intimate Partner Violence Via Daily Diary Surveys: Feasibility, Reporting, and Acceptability. Journal of Interpersonal Violence.

138. White JW, Yuan NP, Cook SL, Abbey A. Ethnic minority women’s experiences with intimate partner violence: Using community-based participatory research to ask the right questions. Sex roles. 2013;69(3-4):226-36.

139. Hartmann WE, Wendt DC, Burrage RL, Pomerville A, Gone JP. American Indian Historical Trauma: Anticolonial Prescriptions for Healing, Resilience, and Survivance. American Psychologist. 2019;74(1):6-19.

140. Ahern EC, Hershkowitz I, Lamb ME, Blasbalg U, Winstanley A. Support and reluctance in the pre‐substantive phase of alleged child abuse victim investigative interviews: Revised versus Standard NICHD protocols. Behavioral sciences & the law. 2014;32(6):762-74.

141. Overlien C, Hellevik PM, Korkmaz S. Young Women's Experiences of Intimate Partner Violence - Narratives of Control, Terror, and Resistance. Journal of Family Violence.

142. DiClemente RJ, Crosby RA, Kegler MC. Emerging theories in health promotion practice and research: John Wiley & Sons; 2009.

143. Follette WC, Houts AC. Models of scientific progress and the role of theory in taxonomy development: A case study of the DSM. Journal of Consulting and Clinical Psychology. 1996;64(6):1120.

144. Van der Hart O, Nijenhuis ER, Steele K. The haunted self: Structural dissociation and the treatment of chronic traumatization: WW Norton & Company; 2006.

145. Heart MYHB. The historical trauma response among natives and its relationship with substance abuse: A Lakota illustration. Journal of psychoactive drugs. 2003;35(1):7-13.

146. Sotero M. A conceptual model of historical trauma: Implications for public health practice and research. Journal of health disparities research and practice. 2006;1(1):93-108.

147. Cusack K, Jonas DE, Forneris CA, Wines C, Sonis J, Middleton JC, et al. Psychological treatments for adults with posttraumatic stress disorder: A systematic review and meta-analysis. Clinical psychology review. 2016;43:128-41.

148. van Minnen A, Hendriks L, Olff M. When do trauma experts choose exposure therapy for PTSD patients? A controlled study of therapist and patient factors. Behaviour research and therapy. 2010;48(4):312-20.

149. Krüger C. Culture, trauma and dissociation: A broadening perspective for our field. Taylor & Francis; 2020.

150. Williams MT, Reed S, Aggarwal R. Culturally informed research design issues in a study for MDMA-assisted psychotherapy for posttraumatic stress disorder. Journal of Psychedelic Studies. 2020;4(1):40-50.

151. Foote B, Smolin Y, Kaplan M, Legatt ME, Lipschitz D. Prevalence of dissociative disorders in psychiatric outpatients. American Journal of Psychiatry. 2006;163(4):623-9.

152. Şar V, Akyüz G, Doğan O. Prevalence of dissociative disorders among women in the general population. Psychiatry Research. 2007;149(1-3):169-76.

153. Karadag F, Sar V, Tamar-Gurol D, Evren C, Karagoz M, Erkiran M. Dissociative disorders among inpatients with drug or alcohol dependency. The Journal of clinical psychiatry. 2005.

154. Seedat S, Stein MB, Forde DR. Prevalence of dissociative experiences in a community sample: relationship to gender, ethnicity, and substance use. The Journal of nervous and mental disease. 2003;191(2):115-20.

155. Dorahy MJ, Brand BL, Şar V, Krüger C, Stavropoulos P, Martínez-Taboas A, et al. Dissociative identity disorder: An empirical overview. Australian & New Zealand Journal of Psychiatry. 2014;48(5):402-17.

156. Brand BL, Sar V, Stavropoulos P, Krüger C, Korzekwa M, Martínez-Taboas A, et al. Separating fact from fiction: An empirical examination of six myths about dissociative identity disorder. Harvard review of psychiatry. 2016.

157. Şar V, Dorahy MJ, Krüger C. Revisiting the etiological aspects of dissociative identity disorder: a biopsychosocial perspective. Psychology research and behavior management. 2017;10:137.

158. Pinderhughes H, Davis R, Williams M. Adverse community experiences and resilience: A framework for addressing and preventing community trauma. 2015.

159. Walters K. Indigenous perspectives in survey research: Conceptualising and measuring historical trauma, microaggressions, and colonial trauma response. Mātauranga taketake: Traditional knowledge Indigenous indicators of well-being: Perspectives, practices, solutions. 2006:27-44.

160. Du Bois WEB. The souls of black folk: Oxford University Press; 2008.

161. Carter RT. Racism and psychological and emotional injury: Recognizing and assessing race-based traumatic stress. The Counseling Psychologist. 2007;35(1):13-105.

162. Bryant-Davis T. Healing requires recognition: The case for race-based traumatic stress. The Counseling Psychologist. 2007;35(1):135-43.

163. Williams MT, Metzger IW, Leins C, DeLapp C. Assessing racial trauma within a DSM–5 framework: The UConn Racial/Ethnic Stress & Trauma Survey. Practice Innovations. 2018;3(4):242.

164. Comas-Díaz L, Hall GN, Neville HA. Racial trauma: Theory, research, and healing: Introduction to the special issue. American Psychologist. 2019;74(1):1.

165. Bryant-Davis T, Ocampo C. Racist incident–based trauma. The Counseling Psychologist. 2005;33(4):479-500.

166. Sanchez-Hucles JV. Racism: Emotional abusiveness and psychological trauma for ethnic minorities. Journal of Emotional Abuse. 1999;1(2):69-87.

167. Bryant-Davis T, Ocampo C. The trauma of racism: Implications for counseling, research, and education. The Counseling Psychologist. 2005;33(4):574-8.

168. Jones SC, Anderson RE, Gaskin-Wasson AL, Sawyer BA, Applewhite K, Metzger IW. From “crib to coffin”: Navigating coping from racism-related stress throughout the lifespan of Black Americans. American Journal of Orthopsychiatry. 2020;90(2):267.

169. Jernigan MM, Daniel JH. Racial trauma in the lives of Black children and adolescents: Challenges and clinical implications. Journal of Child & Adolescent Trauma. 2011;4(2):123-41.

170. Meyer IH, Schwartz S, Frost DM. Social patterning of stress and coping: Does disadvantaged social statuses confer more stress and fewer coping resources? Social science & medicine. 2008;67(3):368-79.

171. Miller GH. Commentary: The trauma of insidious racism. Journal of the American Academy of Psychiatry and the Law. 2009;37(1):41.

172. Difede J, Olden M, Cukor J. Evidence-based treatment of post-traumatic stress disorder. Annual review of medicine. 2014;65:319-32.

173. Zomahoun HTV, Ben Charif A, Freitas A, Garvelink MM, Menear M, Dugas M, et al. The pitfalls of scaling up evidence-based interventions in health. Global health action. 2019;12(1):1670449.

174. Milat A, Lee K, Conte K, Grunseit A, Wolfenden L, van Nassau F, et al. Intervention Scalability Assessment Tool: A decision support tool for health policy makers and implementers. Health Research Policy and Systems. 2020;18(1):1.

175. Indig D, Lee K, Grunseit A, Milat A, Bauman A. Pathways for scaling up public health interventions. BMC Public Health. 2018;18(1):68.

176. Blase K, Fixsen D. Core Intervention Components: Identifying and Operationalizing What Makes Programs Work. ASPE Research Brief. US Department of Health and Human Services. 2013.

177. Underwood E. Screen for childhood trauma triggers debate. American Association for the Advancement of Science; 2020.

178. Loudenback J. California's Surgeon General Readies Statewide Screening for Child Trauma. The Chronicle of Social Change. 2019 September 19, 2019.

179. Finkelhor D. Screening for adverse childhood experiences (ACEs): Cautions and suggestions. Child Abuse & Neglect. 2018;85:174-9.

180. Dowd N, editor Radical ACEs: Building Resilience and Triggering Structural Change. Fla L Rev Forum; 2019: HeinOnline.

181. Foundation RWJ. Policies for Action [Available from: policiesforaction.org.]

1. We use the language used in the original article to define the population. This resulted in some inconsistency in how specific groups are referred to. [↑](#endnote-ref-1)
2. Community Based Participatory Research (CBPR) refers to a set of research principles designed to equitably engage community members in research5. Israel BA, Schulz AJ, Parker EA, Becker AB, Allen AJ, Guzman JR, et al. Critical issues in developing and following CBPR principles. Community-based participatory research for health: Advancing social and health equity. 2017;3:32-5.. Some articles in this review endorsed CBPR principles without reference to CBPR. These articles may be mentioned as endorsing CBPR principles in this appendix. [↑](#endnote-ref-2)
3. Post-traumatic Stress Disorder (PTSD). [↑](#endnote-ref-3)
4. Lesbian Gay Bisexual Transgender Queer/Questioning+ (LGBTQ+) or LGBT refers to an expansive group of gender and sexual minorities. [↑](#endnote-ref-4)
5. Within the Diagnostic and Statistical Manual of Mental Disorders, 5^th^ edition, criterion A refers to a set of experiences that are considered traumas and preconditions for the formation of PTSD. 39. Edition F. Diagnostic and statistical manual of mental disorders. American Psychiatric Association2013. [↑](#endnote-ref-5)
6. Institutional Review Boards (IRBs) [↑](#endnote-ref-6)
